# Direct and indirect effects of a pH gradient bring insights into the mechanisms driving prokaryotic community structures

**DOI:** 10.1186/s40168-018-0482-8

**Published:** 2018-06-11

**Authors:** Daniel R. Lammel, Gabriel Barth, Otso Ovaskainen, Leonardo M. Cruz, Josileia A. Zanatta, Masahiro Ryo, Emanuel M. de Souza, Fábio O. Pedrosa

**Affiliations:** 1 Department of Biochemistry and Molecular Biology, Universidade Federal do Paraná (UFPR), Curitiba, Brazil; 2 Department of Soils and Agricultural Engineer, UFPR, Curitiba, Brazil; 3 Freie Universität Berlin and Berlin-Brandenburg Institute of Advanced Biodiversity Research (BBIB), Berlin, Germany; 4 ABC Research Foundation, Castro, Brazil; 5 Department of Biosciences, University of Helsinki, PO Box 65, 00014 Helsinki, Finland; 6 Department of Biology, Centre for Biodiversity Dynamics, Norwegian University of Science and Technology, 7491 Trondheim, Norway; 7 EMBRAPA Forests, Colombo, Brazil

**Keywords:** pH, Sub-tropical soil, Microbial ecology, 16S rRNA, Illumina sequencing, Soil chemistry, *Bacteria*, *Archaea*

## Abstract

**Background:**

pH is frequently reported as the main driver for prokaryotic community structure in soils. However, pH changes are also linked to “spillover effects” on other chemical parameters (e.g., availability of Al, Fe, Mn, Zn, and Cu) and plant growth, but these indirect effects on the microbial communities are rarely investigated. Usually, pH also co-varies with some confounding factors, such as land use, soil management (e.g., tillage and chemical inputs), plant cover, and/or edapho-climatic conditions. So, a more comprehensive analysis of the direct and indirect effects of pH brings a better understanding of the mechanisms driving prokaryotic (archaeal and bacterial) community structures.

**Results:**

We evaluated an agricultural soil pH gradient (from 4 to 6, the typical range for tropical farms), in a liming gradient with confounding factors minimized, investigating relationships between prokaryotic communities (16S rRNA) and physical–chemical parameters (indirect effects). Correlations, hierarchical modeling of species communities (HMSC), and random forest (RF) modeling indicated that both direct and indirect effects of the pH gradient affected the prokaryotic communities. Some OTUs were more affected by the pH changes (e.g., some *Actinobacteria*), while others were more affected by the indirect pH effects (e.g., some *Proteobacteria*). HMSC detected a phylogenetic signal related to the effects. Both HMSC and RF indicated that the main indirect effect was the pH changes on the availability of some elements (e.g., Al, Fe, and Cu), and secondarily, effects on plant growth and nutrient cycling also affected the OTUs. Additionally, we found that some of the OTUs that responded to pH also correlated with CO_2_, CH_4_, and N_2_O greenhouse gas fluxes.

**Conclusions:**

Our results indicate that there are two distinct pH-related mechanisms driving prokaryotic community structures, the direct effect and “spillover effects” of pH (indirect effects). Moreover, the indirect effects are highly relevant for some OTUs and consequently for the community structure; therefore, it is a mechanism that should be further investigated in microbial ecology.

**Electronic supplementary material:**

The online version of this article (10.1186/s40168-018-0482-8) contains supplementary material, which is available to authorized users.

## Background

In microbial ecology, a key open question is what are the main environmental drivers of microbial community structure, such as the factors involved in the deterministic processes behind community assembly [1, 2]. In soils, pH is usually indicated as the most important driver for soil prokaryotic community structures. Previous studies evaluated microbial communities across pH gradients with high-throughput DNA sequencing and found that both archaeal and bacterial community structures are largely influenced by changes in pH [1, 3–7]. Beyond the microbial community, the influence of pH on the dynamics of many elements in soil is well known, as well as effects on their availability and uptake by plant roots, which may itself influence soil microorganisms [4, 7–9]. It is crucial to improve our understanding of how pH affects microbial communities, because in environmental conditions, pH may be dynamic (e.g., due to root exudates, microbial respiration, and climatic factors) and because controlling soil pH is one of the main practices in agriculture to improve crop production [1, 4, 8, 10].

Little is presently known about the “spillover effects” of pH (indirect effects) on microbial community structure [1, 4], especially in sub-tropical and tropical soils. The indirect effects of pH in soils are wide ranging. pH affects the solubility of different elements, including aluminum (Al^3+^), which can be toxic to plants and microorganisms (Al^3+^ availability decreases with pH, being completely precipitated in pH > 5.5), and also affects the solubility of nutrients (SI 1) [4, 8, 10, 11]. Mineral nutrient availability in soil is mainly studied in an agricultural context and thus focuses on plant-available soluble fractions [8, 12], but these fractions usually also correlate with microbial community structures [4, 13–17]. Phosphorus (P) availability, in the form of phosphate, is optimum at pH 6–6.5 but can be precipitated with iron (Fe), manganese (Mn), and Al in acidic conditions or calcium (Ca) in basic conditions. Additionally, in acidic tropical soils, phosphate can ligate to weathered minerals, such iron and aluminum oxides, rendering it unavailable [8]. Nitrogen (N), sulfur (S), and boron (B) availability (nitrate/ammonium, sulfate, and borate) is optimum between pH 6 and 7.5. The availability of the cations Fe^2+^, Mn^2+^, cupper (Cu^2+^), and zinc (Zn^2+^) decreases with pH, while molybdenum (Mo; molybdate) and chlorine (Cl^−^) availability increases with pH. In acidic conditions, increased Fe and Mn solubility may cause toxicity to plants [8, 18, 19]. Due to these characteristics, plant growth is usually optimal in the pH (CaCl_2_) range of 5.5–6.5 [8, 10].

Although overall bacterial diversity is usually also highest in this pH range [1, 3, 6, 20], the optimal ranges of pH and nutrients for most environmental microorganism species are still largely unknown [4, 9, 21]. Some studies investigating the interaction of plants and microorganisms, such as the symbiosis between rhizobia and leguminous plants, demonstrated that Ca, P, Fe, and Mo stimulate these bacteria and that their optimal pH was also near 6 [11, 22–24]. Other examples of the effects from pH-driven changes in soil nutrient availability on bacterial species or communities are summarized in Additional file 1.

Due to the effects of pH on elemental solubility, a common practice in agriculture consists of applying lime (Ca and Mg carbonates) in order to increase soil pH, simultaneously increasing the concentrations of Ca and Mg [8, 10]. This leads to increased plant growth that usually results in higher output of plant exudates, roots and litter decomposition, and consequently higher soil organic matter (SOM) [8, 25]. Moreover, increased litter accumulation will result in increased nutrient cycling on the soil surface, as bigger plants will extract more nutrients from different soil depths that are then decomposed and mineralized on the surface (e.g., K and NO_3_) [26, 27]. On the other hand, plants growing in acidic soils are not usually smaller but also have altered metabolisms and different biomass composition—for example, some soybean varieties produce root organic exudates (e.g., malate) to reduce toxic effects of Al [28, 29]. Differing plant biomasses and root exudates resulting from soil pH effects can subsequently influence microbial community structures, since they are usually responsive to different carbon sources and quantities [7, 30].

**Fig. 1:**
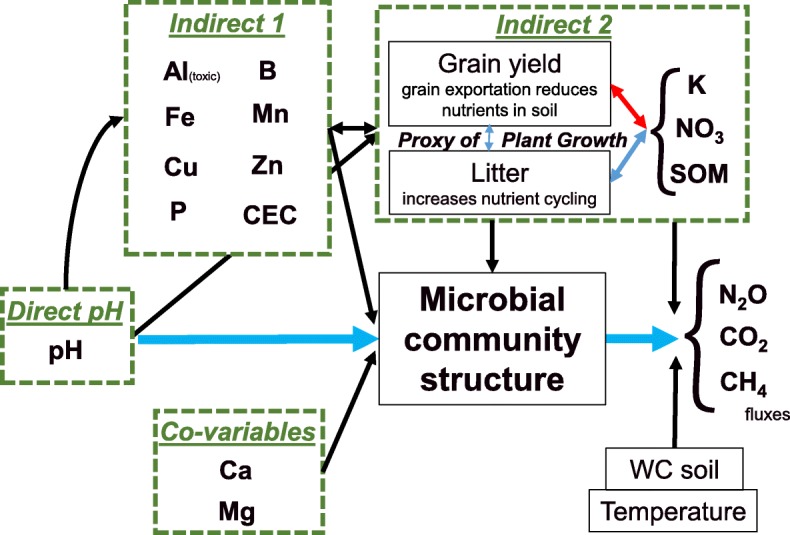
Simplified theoretical diagram of expected interactions in the pH range between 4 and 6 with the microbial community structure (full diagram available in Additional file 1). The direct effect of pH is expected to be the biggest driver of microbial community structure. In this study, the pH gradient was produced by liming application, thereby producing quantifiable co-variables (Ca and Mg). The indirect effects are the “spillover” effect of the pH in the other soil and plant variables. Indirect effect 1 is mainly related to the solubility of elements (Al, B, Fe, Mn, Cu, Zn, P) and the cation exchange capacity (CEC), while indirect effect 2 is related to these effects on plant growth and consequently on soil organic matter (SOM) and nutrient cycling (e.g., K and nitrate, NO_3_). Temperature and soil water content (WC) are considered in this diagram only for the survey day of greenhouse fluxes (as a proxy for microbial activity)

Because of all these factors, pH can have direct and indirect effects on soil microbiota that are summarized in a conceptual model (Fig. 1). Moreover, all these effects can influence not only microbial community structure but also microbial physiology and activity. This can have consequences for biogeochemical cycles, changing fluxes of CO_2_, and the greenhouse gases (GHG) CH_4_ and N_2_O. The flux of these gases, especially CO_2_ and N_2_O, in soils generally increases with increasing pH [1, 31].

Direct and indirect effects of pH are usually well described in the soil fertility research field but are rarely addressed in microbial ecology [8]. While pH is frequently measured, the other elements and factors that usually co-variate with it (e.g., Ca, Al, Fe, Mn) are often ignored. Also, in most previous studies, pH co-varied simultaneously with multiple confounding factors, such as land use, soil management (e.g., fertilizers, lime, and pesticides usage), plant cover, and/or edapho-climatic conditions (e.g., soil type, clay content and type, precipitation, and temperature) [3, 14, 32, 33]. For example, in a study comparing bacterial community structure shifts due to land-use change in south Amazonia, pH co-varied with plant cover, liming, and other agricultural practices, making it difficult to disentangle the most important driver [14]. On the other hand, two studies using lime (Hoosfield acid strip at Rothamsted Research, UK) and fertilizer (Park Grass experiment, UK) gradients were better able to separate the effects of pH on microbial communities, since confounding factors were minimized [1, 4]. Furthermore, the latter study suggested that factors that co-varied with pH should be investigated in greater detail (e.g., mediation of nutrient availability). Thus, the application of lime or fertilizer gradients in areas with identical land-use permit increased control over edapho-climatic conditions, chemical inputs, and plant cover to allow for improved investigations of pH and co-varying factors on microbial communities [1, 4].

The aim of this study was to evaluate an agricultural pH gradient (from 4 to 6.2, a typical range for tropical farms), quantifying changes in archaeal and bacterial community structure and investigating relationships with soil parameters, according to the conceptual model (Fig. 1). We hypothesized that in addition to the direct effects of pH, indirect effects are also important drivers for prokaryotic community structure.

## Methods

### Study area and experimental design

Soils were sampled from a pH gradient in the agricultural region of the “Campos Gerais” in southern Brazil, which is one of the biggest producers of soybean (*Glycine max*), corn (*Zea mays*), and wheat (*Triticum aestivum*) in the country [34]. This area was under the experimental management of the ABC Research Foundation, where a typical no-till farm was identified with soil pH around 4 (coordinates 24° 40′ 34.7″ S and 50° 26′ 52.5″ W, 748 m amsl) and a liming experiment produced a gradient of pH (CaCl_2_) from 4 to 6.2 (the typical pH range of farms in tropical regions). It is estimated that 31.8 million hectares are cultivated in Brazil with similar no-till techniques and soil pH ranges [34]. The soil was identified as a Red Latosol (Oxisol—the most common soil type in tropical crop regions), with clayey texture. The climate in the region is mesothermal warm summer (Cfb, by the Kӧppen–Geiger classification) [35], with 18.7 °C annual average temperature and 1335 mm annual precipitation.

Direct and indirect effects of pH on soil prokaryotic community structures are usually difficult to disentangle due to co-variables and confounding factors. Here, we surveyed a previously established liming experiment that produced a pH gradient with quantifiable co-variables (Ca and Mg). The experiment was composed of four lime doses (0, 2250, 4500, and 6750 kg ha^− 1^ of Ca and Mg carbonates) applied to nine replicate plots of 9 × 7 m in blocks. Within the nine replicate plots, three different lime brands were used, reducing bias. Thus, in the analysis, we assumed a nested design, considering the brands as fixed effects in each dose. The experiment was maintained following typical crop management and rotation from the region, and all the plots received exactly the same treatments (Additional file 2: Table SI2 A), except the lime doses described above. The soil was sampled in April 2014 (5 years after the lime application), 3 weeks after soybean harvest, allowing the use of the grain yield and litter quantification as proxies for plant growth.

### Sampling

First, one 32-cm-diameter ring was inserted into the soil of each experimental plot, and the litter (dead debris from previous crop harvests, mainly soybean harvested in summer 2014 and oats from the previous winter season) was removed from the soil surface and placed in paper bags for quantification. One hour later, greenhouse gas (GHG) measurements were performed using these rings as bases and 20-l static chambers for gas accumulation [36, 37]. The chambers were coated with thermal insulation to avoid an increase of temperature during GHG sampling and had a ventilator inside to homogenize the air samples. Then, gas samples were collected with 20-ml polypropylene syringes at 0, 5, 10, and 20 min and stored in hermetic glass vessels (Labco Exetainer® Vials) until analysis in the same week for quantification of CO_2_, N_2_O, and CH_4_ [36–38]. This system was previously tested with standard gases, and no leaking was observed. In parallel, air temperatures inside the chambers were measured.

Then, soil samples were collected from a 0–10-cm depth. Five soil cores were surveyed in each plot and homogenized, and approximately 200 g was stored in aseptic plastic bags. All the tools used were previously disinfected with ethanol 80%. Aliquots for DNA and nitrate extractions were placed in 15-ml sterile conical tubes on ice and later stored at − 20 °C until analyzed.

### Chemical analysis

Soil samples were processed according to the standard methods for Brazilian tropical soils proposed by the IAC [12]. First, samples were sieved to 2 mm and air dried. The pH values were measured in H_2_O and in 0.01 M CaCl_2_ (across the manuscript, we report pH CaCl_2_ values, since it is the most stable pH used in soil analysis [8]). The exchangeable cations (K^+^, Ca^2+^, and Mg^2+^) and available P-phosphate were extracted using ion exchange resins; Al^3+^ was extracted with KCl 2 M, the trace elements Cu^2+^, Fe^2+^, Mn^2+^, and Zn^2+^ were extracted by diethylenetriaminepentaacetic acid (DTPA) and triethanolamine, and B was thermally extracted in water and analyzed according to Cantarella et al. [12]. K, Ca, Mg, Al, Cu, Fe, Mn, and Zn were analyzed by an atomic absorption spectrophotometer (Analytik Jena, contrAA300), and P and B were analyzed by the ammonium molybdate method and by the azometin-H method, respectively, and read spectrophotometrically (Micronal, AJX 1600) [12]. Cation exchange capacity (CEC) was estimated by summing H, Al, Ca, Mg, and K. Nitrate was extracted by adding 4-g frozen soil to 40 ml of 2 M KCl, agitated for 1 h, and filtered [38] and analyzed spectrophotometrically (Tecan, Infinite M200 PRO) [39].

### Gas analysis

Gas samples were analyzed by gas chromatography (Thermo Scientific, GC TRACE 1310), and the concentration of CO_2_ and CH_4_ were determined by a flame ionization detector (250 °C) and N_2_O by a Ni-electron capture detector (320 °C). Injector was setting to 250 °C and Porapak columns to 70 °C of temperature. The GC was calibrated with standard gases (White Martins, Praxair), and standard gases were read periodically (between every 20 samples), as quality control. The gas molar volume (Vm) was corrected for the headspace chamber air temperature (K) as measured at sampling time. And the gas fluxes (*f*) were calculated by each gas considering the change in gas concentration in the chamber during the incubation time (Δ*C*/Δ*t*), the chamber volume (*V*), the soil area covered by the chamber (*A*), and the molecular weight of the gas (*m*), by the equation: *f* = Δ*C*/Δ*t* × V/A × *m*/Vm [36–38].

### DNA extraction, 16S rDNA amplification, and sequencing

The 15-ml frozen soil samples were ground in mortars in liquid nitrogen to improve homogeneity and lysis efficiency. Aliquots (250 mg) were then used for extraction with the Power Lyzer Soil DNA Isolation Kit (MOBIO laboratories, Inc.), and DNA concentration was analyzed spectrophotometrically (Thermo Fisher Scientific, Nanodrop). Later, the V4 region of the 16S rRNA was amplified by PCR using 1 μl of the DNA extracts (49 ± 9 ng of DNA) and the KlenTaq Master Mix 1X (Sigma) and the primers 515F and 806R [40]. PCR conditions were 94 °C for 3 min and 18 cycles of 94 °C for 15 s, 50 °C for 30 s, and 68 °C for 60 s, followed by 68 °C for 7 min. PCR was performed in triplicate for each sample, and then, the amplicons were merged in equal volumes, quantified fluorometrically (Thermo Fisher Scientific, Qubit), pooled, purified using the PureLink PCR Purification Kit (Invitrogen), and sequenced with the v2 Reagent Kit (500 cycles PE) in the MiSeq platform (Illumina, MiSeq), following the manufacturer’s instructions [40]. Sequences were deposited in the NCBI Genbank (BioProject PRJNA413794).

### Sequence analysis

Sequencing data were analyzed with the QIIME pipeline [41]. Sequences were quality filtered and identified according to the SILVA 123 database [42, 43]. Since the Illumina output ranged from ~ 4000 to 100,000 reads per sample, they were re-sampled to 31,000 reads per sample, allowing the diversity comparisons [44]. For four samples, sequencing yielded less than 31,000 reads, and so, these samples were excluded from the analysis (analyses were applied for 32 samples). QIIME output was exported for further analysis.

### Statistical analysis

Statistical analyses were first performed using the R software [45], and the package Vegan was used to calculate the Simpson diversity index [46]. Soil data were analyzed by ANOVA and ranked according to the Tukey post hoc test (*P* < 0.05) (SI 2). The operational taxonomy units (OTUs; previously identified by the QIIME pipeline at phylum and genus levels) were correlated with the chemical data (Pearson and Spearman correlation indices). Also, unweighted UniFrac distances previously obtained from QIIME were used for ordination using PCoA and further correlated with the environmental parameters using Vegan envfit function [46].

### Hierarchical modeling of species communities (HMSC)

We then analyzed the data with HMSC [2], an approach that belongs to the class of joint species distribution models (JSDM; [47]), using R and Mathlab. We considered four groups of explanatory variables based on the conceptual model (Fig. 1). The first group (G1 = direct 1) of variables consisted solely of pH, as this variable was our main focus. The second group (G2 = direct 2) consisted of the effects of Ca and Mg, the variation in which we considered to be a direct consequence of the lime application (co-variables). The third group (G3 = indirect 1) consisted of those variables that we expected to be primarily influenced by changes in pH: Al, Fe, Mn, Cu, Zn, P, B, and CEC. The fourth group (G4 = indirect 2) consisted of those variables that we expected to be secondarily related to pH: soybean yield and litter (both proxies of plant growth), soil organic matter (SOM), K, and NO_3_. In order to measure the influences that are beyond those explained by pH, we regressed the variables in groups G2, G3, and G4 against pH and used the residuals from these models as explanatory variables. Further, due to the experimental design (*n* = 32), we reduced the number of explanatory variables to avoid overfitting. To do this, we run principal component analysis and included as explanatory variables only the two first principal components of the variables in groups G3 and G4. Thus, the full model consisted of seven explanatory variables: pH (G1), Ca and Mg (G2), and two variables representing G3 (principal components G3_1 and G3_2) and G4 (G4_1 and G4_2).

Due to the zero-inflated nature of the data (absences) and the high number of OTUs involved, we constructed two models, one for presence–absence (assuming probit link function and Bernoulli distribution), and another one for abundance (assuming normal distribution for log-transformed and centered data). In the presence–absence model, we included only those 248 OTUs that were both present and absent in at least 10 samples and thus showed substantial variation in occurrence. In the abundance model, we included only those 271 OTUs that were present in all samples and thus showed variation in terms of abundance but not occurrence. To quantify the influences of variables other than pH, we also considered four reduced models containing subsets of full model predictors: (i) G1 only, (ii) G1 and G2, (iii) G1, G2, and G3, and (iv) G1, G2, and G4. We examined the predictive powers of these models through fivefold cross-validation (Additional file 3: Table SI3 A, B). We thus randomly split the 32 data points into five groups and predicted the data for each group by a model fitted to a subset of the data from which the focal group was excluded. We assessed the predictive performance of the models separately for each species, by Tjur *R*^2^ [48] for the presence–absence models and by correlation for the abundance models. We averaged the species-specific values to obtain an overall measure of model performance.

Additionally, to examine if the responses of the OTUs to the explanatory variables are structured by phylogeny, we included a phylogenetic correlation matrix (generated based on the QIIME phylogenetic tree) into the HMSC analyses. To assess possible co-occurrence patterns among the OTUs that cannot be attributed to their responses to the environment, we also included a community-level random effect implemented through a latent variable approach [2].

### Random forest (RF)

The abundance of each OTU was modeled according to the environmental predictors using the machine-learning algorithm RF, which is independent of data distribution [49]. RF algorithms are able to evaluate the relative importance of predictors that are highly correlated to each other (cf. multicollinearity), thereby improving confidence in determining which predictors are affected more strongly to the response variable [50, 51]. Using the approach by Hapfelmeier and Ulm [52], statistically significant predictors were selected with a permutation approach (*P* = 0.05, trees = 500, and permutations = 400). If none of the predictors were significant, no model was produced for the response variable (OTUs). Model performances were measured as variance explained (*R*^2^) and validation score, and OTUs that had a validation score lower than 0.1 were excluded from the “Results” section (but are shown in Additional file 4).

## Results

**Fig. 2:**
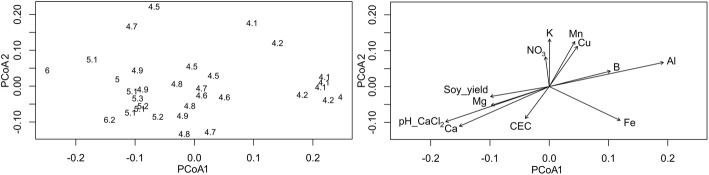
Principal coordinate analysis (PCoA) based on unweighted UniFrac distance depicting the prokaryotic diversity according to **a** each sampling point (represented in the plot by its pH CaCl_2_ values) and **b** the biplot of the significant environmental parameters (*P* < 0.1)

The direct and indirect effects of a pH gradient on the soil prokaryotic community structure were evaluated. A lime gradient produced alterations in the soil pH (ranging from 4.1 to 6.2) that caused changes in soil chemistry and shifts on the prokaryotic community structures. Some effects were observed at Phylum level (Table 1 and Additional file 5), including two archaeal phyla and 17 bacterial phyla that correlated with pH (e.g., *Bacteroides p* 0.65, *Hydrogenedentes p* 0.74, and *WD272 p* − 0.78; *P <* 0.001). But the strongest effects were observed at genus level, as will be described in Fig. 2 and Table 2.

**Table 1:** Spearman (*ρ*) Correlation indexes (or Pearson when indicated “*r”*), between the soil pH CaCl_2_, Ca and Mg with analyzed parameters and the relative abundance of archaeal and bacterial phyla (only significant values are shown: *r* or *ρ* >0.4 or <-0.4 and *P*<0.05)

Variable	pH (CaCl_2_)	Ca	Mg
*Soil Chemistry*			
pH (H_2_O)	0.98^a^	0.94	0.72
Ca	0.95	1.00	
Mg	0.72	0.65	1.00
Al	-0.98	-0.94	-0.70
Fe	-0.51	-0.44	
Mn	-0.51	-0.53	-0.41
Cu	-0.45 (*r*)	-0.51 (*r*)	
B	-0.54 (*r*)	-0.44	-0.46
P			0.48
CEC	0.52 (*r*)	0.69 (*r*)	0.56 (*r*)
*Soy Yield*	0.49	0.45	
*Simpson diversity index*	0.73	0.60	0.59
*Archaeal Phyla*			
Euryarchaeota	0.41	0.44	
Woesearchaeota	0.59 (*r*)	0.51 (*r*)	
*Bacterial Phyla*			
Bacteroidetes	0.69 (*r*)	0.54	0.48
OP3	0.52 (*r*)	0.46 (*r*)	0.52
SR1	0.45 (*r*)		
Gemmatimonadetes	0.49 (*r*)	0.41	
Hydrogenedentes	0.74	0.76 (*r*)	0.64
Latescibacteria	0.53	0.49	
Lentisphaerae	0.44	0.45	
Microgenomates	0.76 (*r*)	0.70 (*r*)	0.58
Nitrospirae	0.55	0.51	0.55
Omnitrophica	0.58 (*r*)	0.49 (*r*)	0.49
Parcubacteria	0.43 (*r*)	0.47 (*r*)	
Planctomycetes	0.65 (*r*)	0.66 (*r*)	0.48
Proteobacteria	0.40 (*r*)	0.45 (*r*)	
Verrucomicrobia	-0.61	-0.61	
WCHB1.60		-0.42	
WD272	-0.83 (*r*)	-0.73	-0.64
Unclassified Bacteria	-0.44	-0.47	

^a^We reported Spearman (*p*) correlations since it fitted better for most of the data and Pearson (r) in the cases it fitted better (full data is available in Additional file 3)

**Table 2:** Selection of the 15 most abundant OTUs (average of all samples) at genus level and potentially beneficial genera that correlated with pH and selected soil parameters (Spearman correlation, *p*, only significant values are shown, *P* < 0.05 and *p* > 0.4 or <− 0.4), and also the *R*^2^ of the random forest (RF) models for the same parameters. The highest correlation values and RF-R^2^ in each line, positive or negative, are depicted in bold (the full list of OTUs and their correlations is available in Additional file 6)

OTU	Phylum	Genus	Frequency (%)	pH CaCl_2_	Ca	Al	Fe	Cu	pH CaCl_2_	Ca	Al	Fe	Cu
				(*p*)	(*p*)	(*p*)	(*p*)	(*p*)	(*R*^2^)	(*R*^2^)	(*R*^2^)	(*R*^2^)	(*R*^2^)
15 most abundant OTUs correlated with pH													
OTU879	*Proteobacteria*	*Variibacter*	8.097	− 0.45		0.48	**0.51**		0.03		0.04	**0.19**	< 0.01
OTU812	*Proteobacteria*	*Bradyrhizobium*	2.938	− 0.49^a^			**0.51**		0.02	< 0.01	0.05	**0.23**	
OTU787	*Proteobacteria*	Uncultured	2.720	**− 0.48**		0.47	0.42		0.07	0.01	**0.13**	0.04	
OTU290	*Actinobacteria*	Uncultured	2.507	− 0.60	− 0.48	**0.63**	0.55		0.16	0.03	**0.15**	0.12	
OTU87	*Acidobacteria*	Uncultured	2.273	0.74	0.75	**− 0.76**		− 0.43	0.15	0.18	**0.29**	< 0.01	0.01
OTU35	*Acidobacteria*	Uncultured	2.224	**− 0.80**	− 0.77	**0.80**	0.46		0.19	**0.22**	0.19	0.02	< 0.01
OTU1353	*Verrucomicrobia*	*C. xiphinematobacter*	2.087	**− 0.47**		**0.47**			0.10	0.05	**0.15**	0.01	< 0.01
OTU1344	*Verrucomicrobia*	Uncultured	2.052	**− 0.61**	**− 0.61**	0.55		0.52	**0.18**	**0.18**	0.01		0.08
OTU1287	*Proteobacteria*	*Acidibacter*	1.795	**− 0.65**	**− 0.65**	0.63			**0.15**	0.10	0.15		0.02
OTU139	*Actinobacteria*	*Acidothermus*	1.763	− 0.57	− 0.46	**0.62**	0.61		**0.19**	0.03	0.13	0.15	
OTU1117	*Proteobacteria*	*Haliangium*	1.676	**0.56**	0.50	− 0.53			0.16	0.06	**0.15**	< 0.01	< 0.01
OTU1342	*Verrucomicrobia*	Uncultured	1.662	**− 0.71**	− 0.67	0.66			**0.14**	0.12	0.13	0.01	< 0.01
OTU53	*Acidobacteria*	Uncultured	1.547	− 0.56	**− 0.57**	0.54		0.48	0.06	0.05	0.04	0.01	**0.10**
OTU84	*Acidobacteria*	*Ambiguous_taxa*	1.445	0.72	**0.73**	**− 0.73**		− 0.57	0.18	0.08	**0.20**		0.10
OTU287	*Actinobacteria*	Uncultured	1.283	**− 0.53**	− 0.42	**0.53**			0.11	0.05	**0.13**	0.03	0.01
Potentially beneficial genera^b^													
OTU858	*Proteobacteria*	*Rhizobium*	0.085	0.55	0.43	− 0.54	**− 0.65**		0.11	0.06	0.04	**0.18**	
OTU852	*Proteobacteria*	*Mesorhizobium*	0.048	**0.73**	0.71	− 0.68	− 0.49	− 0.49	**0.24**	0.18	0.08	0.02	0.02
OTU1032	*Proteobacteria*	*Herbaspirillum*	0.003	**− 0.52**	− 0.47	0.51			**0.11**	0.08	0.08		< 0.01

^a^We reported Pearson (*r*) for *Bradyrhizobium* with pH, since it fitted better (full data in Additional file 6)

^b^OTUs that may potentially promote plant growth [53, 54]

### Soil chemistry, plant yield, and gases

The pH gradient caused alterations to several soil chemical attributes (Table 1 and Additional file 2). The direct effect of the liming increased the pH CaCl_2_ (from 4.2 ± 0.3 in the control to 5.3 ± 5 in the highest lime dose) and the concentration of the co-variables Ca (from 22.9 ± 10.9 in the control to 81.4 ± 31.8 cmolc dm^3^ in the highest lime dose) and Mg (from 9.6 ± 8.0 to 21.3 ± 14.8 cmolc dm^3^). Indirect effects of the pH were also detected, accordingly to the correlation between pH with the different variables (*P* < 0.01): Al (*p* − 0.98), Fe (*p* − 0.51), Mn (*p* − 0.51), Cu *(r −* 0.45), B (*r* − 0.54), CEC (*r* 0.52), and soybean yield (*p* 0.49) (Table 1). No significant correlations were observed for litter quantity, nitrate, and gas emissions (*P* < 0.05). Perhaps, future studies using litter traps and temporal survey of the gases could bring better correlations. Litter quantity among all plots was 78.8 ± 31.7 g m^− 2^, nitrate 6.4 ± 1.1, CO_2_ flux 107 ± 89 mg m^2^ h^− 1^, CH_4_ flux − 7 ± 19 μg m^2^ h^− 1^, and N_2_O flux 13 ± 60 μg m^2^ h^− 1^, but no statistical difference was observed between the pH ranges or lime doses (*P* < 0.05, Additional file 2: Table SI2 B). However, some OTUs correlated with gas fluxes (mostly *Proteobacteria*), of which 34 OTUs correlated with CO_2_, 46 with CH_4_, and 32 with N_2_O (Additional file 6).

### pH effects on the community structures

Community structures were highly modulated by pH (Fig. 2), as indicated by the PCoA ordination of the samples across the first axis of the PCoA analysis (PCoA1, Fig. 2). The indirect effects of pH were then indicated by the sample ordination across the second axis (PCoA2) and both supported by the *envfit* algorithm coefficients (*P* < 0.05) with pH CaCl_2_ (*r*^2^ 0.85), the co-variates Ca (*r*^2^ 0.75) and Mg (*r*^2^ 0.26), and the indirect effects 1: Al (*r*^2^ 0.88), B (*r*^2^ 0.26), Cu, (r^2^, 0.31), Mn (*r*^2^ 0.37), and Fe (*r*^2^ 0.49), and the indirect effects 2: soy yield (*r*^2^ 0.31), NO_3_ (*r*^2^ 0.15), K (*r*^2^ 0.37), and CEC (*r*^2^ 0.20) (variables statistically not significant were not reported). Additionally, the Simpson diversity index positively correlated with pH (*p* 0.73, *P* < 0.01) (Table 1).

Further analysis revealed that 493 out of 1374 OTUs (at genus level) were highly correlated with pH, but some were correlated also with the indirect effects (Additional file 6). Among the 15 most abundant OTUs correlated with pH, 11 of them correlated equally or better with the indirect effects (e.g., Ca, Al, Fe, and Cu) (Table 2). For example, *Variibacter* and *Bradyrhizobium* (relative abundances of 8.1 and 2.9%) had negative correlations with pH, but correlated better with Fe, as supported by the RF analysis (Table 2). The same pattern occurred for bacteria potentially beneficial to plants. *Rhizobium*, *Mesorhizobium*, and *Herbaspirillum* (that can promote plant growth [53, 54]) had correlation not only with pH but also with Ca and Al, and *Rhizobium* was correlated better with Fe (Table 2).

### HMSC analysis

**Fig. 3:**
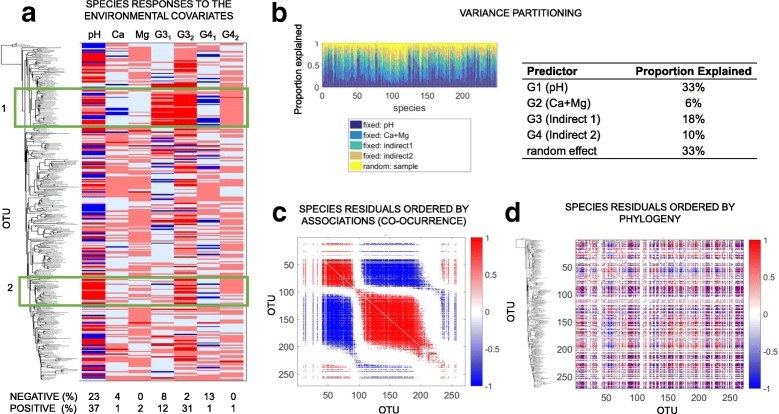
Panels highlighting the main HMSC results based on the abundance model (details in Additional file 3). **a** HSMC-based estimates of species responses to the environmental covariates. The OTUs were ordered by their phylogeny (high-resolution tree in Additional file 7), as illustrated by the plots. Positive and negative responses, based on posterior mean, are shown in red and blue, respectively. The darker red and blue colors corresponding to cases with strong statistical support (posterior probability at least 95%), and the percentages of such OTUs are given on the bottom of the panel. The areas highlighted by the green rectangles are discussed in the text (1, *Deltaproteobacteria* and 2, *Actinobacteria*). **b** Variance partitioning of the species responses to the environmental covariates. Panels **c** and **d** show the HMSC-based estimates of species residual (after accounting for influences of covariates) associations. In panel **c**, the species have been ordered in a way that best show clusters of associated OTUs, whereas in panel **d**, they have been ordered by the phylogeny (as illustrated in the plots). Red (respectively, blue) entries show OTU pairs for which the residual association is positive (respectively, negative) with at least 95% posterior probability

The data were further explored using HMSC. The cross-validation exercise of the models showed that the presence–absence model had little predictive power (*R*^2^ 0.06), whereas the abundance model had better predictive power (*R*^2^ 0.29), so the results are shown for this model (Fig. 3; see Additional file 3 for the presence–absence model). Variance partitioning indicated that the pH was the most important explanatory variable (33%) and the group G3 (indirect 1) was the second most important variable (18%), which is in accordance with the cross-validation results (*R*^2^ 0.28, when including both variables). A large proportion of OTUs obtained strong statistical support for either a positive (37 OTUs) or a negative response to pH (23 OTUs) and also to the variables from the group G3 (indirect 1; 43 positive and 10 negative responses) and G4 (indirect 2; 13 negative and 2 positive responses) (Fig. 3). Of these, 39 OTUs responded more strongly to indirect effects than to the pH itself, including 17 *Chloroflexi* and 8 *Proteobacteria* (Additional file 3: Table S3 C)*.*

There was a strong phylogenetic influence on how the species responded to the explanatory variables. The parameter *ρ*, that measures the strength of the phylogenetic signal in the HMSC model (varying from 0 to 1) [2], was 0.68 (0.54–0.83) for the presence–absence model and 0.99 (0.97–1.00) for the abundance model (95% confidence interval). This effect is shown in Fig. 3, e.g., the OTUs belonging to rectangle 1 (some *Proteobacteria*) responded positively (increase in abundance) to group G3 (indirect 1), while rectangle 2 (some *Actinobacteria*) responded positively to pH. The community-level random effect included in the model captured also residual co-occurrence patterns among the OTUs that were not explained by the environmental covariates (Fig. 3c). The residual co-occurrences were unrelated to phylogeny, as the association network ordered by phylogeny lacks a clear structure (Fig. 3d).

### RF analysis

**Fig. 4:**
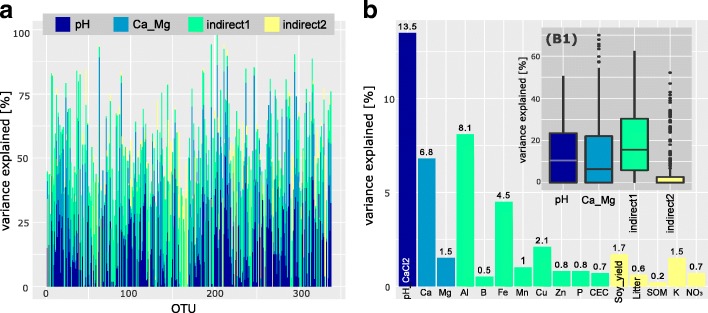
Summary of the RF models for each OTU that had a validation score higher than 0.1; the overall averages of variance explained *R*^2^ and validation score were 0.45 and 0.23, respectively (full data is available in Additional file 4). **a** Variance explained (%) by each category of the predictors in our conceptual model for each OTU. **b** Variable importance of each predictor that was averaged across all the models (the numbers are the mean values in %); the box plot (B1) summarizes the distributions of all the predictors according to the conceptual model categorization across the OTUs (Fig. 1)

Lastly, the OTUs were modeled using RF. From the 1374 OTUs, 810 were successfully modeled based on the best model selection (*P* = 0.05), resulting in overall mean variance explained of 0.32 and mean validation score of 0.12 (Additional file 4). After screening the models to validation score higher than 0.1, 338 OTUs were selected, resulting in overall mean variance explained of 0.45 and mean validation score of 0.23 (Fig. 4). The top five individual predictors were, in order of importance, the following: pH with 13.5%, Al 8.1%, Ca 6.8%, Fe 4.5%, and Cu 2.1%. We also built RF models for the greenhouses fluxes, CO_2_, N_2_O, and CH_4_, but neither pH nor individual OTUs or Simpson diversity indices were significant predictors.

## Discussion

It is well known that pH influences the growth and diversity of prokaryotes [1, 4–6, 20]. However, many other factors in soil are also influenced by pH that may also indirectly affect microbial communities, and this issue is still unclear and not explicitly investigated [1, 4]. Thus, the investigation of these aspects is highly relevant for microbial ecology and provides insight into the mechanisms driving prokaryotic community structures in soils. Our results indicated a clear effect of pH on the microbial communities (Table 1 and Fig. 2), as previously reported [1, 3–6, 20] but, furthermore, indicated that indirect effects and co-variables also played a significant role in the microbial community assembly (Figs. 3 and 4). Interestingly, we also detected an influence on the phylogenetic structure of the community, related to both the direct and indirect effects of pH, suggesting a certain level of phylogenetic niche conservatism (Fig. 3).

Our data indicate that diversity increases with pH changing from 4 to 6 (Spearman correlation, *p* 0.73, Table 1), which is in agreement with several studies that showed maximum values of prokaryotic diversity in the pH range 6–7 [1, 3, 6, 20]. In this pH range, the highest overall availability of soil nutrients is expected, while metal (i.e., Al, Fe, Mn) concentrations are typically non-toxic [8, 18]. However, up to now, only a few studies have suggested this link between the indirect effects of pH on soil elements with microbial community structures [4, 14].

Moreover, our data point to a strong influence of pH on the community structure indirectly throughout effects on elemental availability (Al, Fe, Cu, Mn, Zn, P, and B; Figs. 2, 3, and 4). Previous studies in tropical soils that investigated shifts in bacterial community structures due to land-use change also detected similar correlations [15, 32]. In these studies, changes in the microbial community structures were directly correlated with pH, as well to elemental availability (e.g., Ca, Mg, P, Al, Fe, Cu, B, and Zn). However, during land-use change, simultaneously, changes occur, such as plant cover and applications of lime and fertilizers, masking indirect relationship between pH and the co-variates [14]. Another study in New Zealand evaluated multiple factors in soils, and their results also indicated that some pH effects on bacterial community structures were parallel to the P, Al, Cu, and Mg effects [17]. In our study, with confounding factors minimized, we could clearly observe the same trends, where some taxa (e.g., *Acidobacteria* and *Proteobacteria*) also shifted simultaneously (e.g., in response to pH and Al). Additionally, in the Park Grass experiment in UK, a pH gradient that had confounding factors minimized, community structures were linked to pH, P, C, and N, and the authors suggested that the effect of pH on nutrient availability was the most probable mechanism, in line with our results [4].

In a further analysis of the variables that impacted specific groups in a stronger way than directly pH in our dataset, the HMSC models indicated 39 OTUs, of which 17 were *Chloroflexi* and 8 *Proteobacteria* (SI 3-C). RF indicated that these OTUs were mostly related to Al, Fe, Zn, and Cu (Additional file 4). This is in agreement with previous studies that report some *Proteobacteria* and *Chloroflexi* tolerant to heavy metals and that soil communities respond to metal gradients (such Fe, Zn, and Cu) in soil [13, 16, 17]. Interestingly, the phylogenetic closely related OTUs *Varibacter*, *Bradyrhizobium*, and *Rhizobium* were highly correlated with Fe (Table 2), and a recent review highlighted the diverse and complex mechanisms that rhizobia uses for Fe homeostatic control, including tolerance to high levels [55]. We also detected indirect effects of pH on plant growth and litter nutrient cycling (e.g., K and NO_3_) that influenced microbial community structure (Fig. 1, indirect 2) [27]. A total of 35 OTUs (including 11 *Actinobacteria* and 14 *Proteobacteria*; Figs. 3 and 4) were found to change under these influences. Among these OTUs, one example is *Azospirillum*, a well-known genus that contain representatives of plant growth-promoting bacteria associated to rhizosphere [53, 54].

Additionally, we found that the microbial community structures influenced by the direct and indirect effects of pH were also influenced by phylogenetic patterns. It could be that closely related organisms, due to similarity on basic structural and physiological processes, are more likely to react similarly to the direct and indirect effects of pH [56, 57], as shown to some *Actinobacteria* and *Proteobacteria*, respectively (Fig. 3). After accounting for the direct and indirect effects, the communities were better assembled by association with other OTUs (co-occurrence) than with phylogeny (Fig. 3c, d), indicating that the detected phylogenetical patterns occurred only for the pH-related factors. This result is in line with a recent study that found that communities in more acidic soils are driven by deterministic process and linked to phylogenetic relationships, while soils close to neutral pH are more influenced by stochastic processes not related to phylogeny [6].

Although this phylogenetical pattern was observed for some groups of OTUs in our data, it is still hypothesized that a high degree of variation to pH exists even at species/strain levels [1] and not well resolved by 16S rRNA gene sequence-based analysis. For example, it is known that during isolation and screening of symbiotic *Bradyrhizobium* isolates, a wide variation of genotypes growing in different pH ranges can be found, with a generally expected predominance in neutral pH ranges [23, 24]. In our study, *Bradyrhizobium* (including environmental genotypes) had higher relative occurrence in low pH, as also reported in a European soil [4]. This is also in accordance with the result that different groups of OTUs identified within *Actinobacteria* may benefit from higher (Fig. 3) or lower pH (Table 2). These examples illustrate that not always taxonomic groups (e.g., phylum/genus) unequivocally correlate to specific pH ranges. Even though correlations are sometimes observed, usually, they are highly variable when comparing different study sites [1, 4]. For example, usually, *Acidobacteria* is suggested as negatively correlated with pH, but in our study, as well as others in Europe and the Amazon, variations were observed [1, 32].

Also, our data strongly support that OTUs should be better investigated regardless not only to the direct effects of pH but also to the indirect effects of pH which are the main drivers for their occurrence. In recent years, there has been a large increase in the number of studies correlating prokaryotic community structures to pH, so we suggest that it is timely a call to more studies focusing to disentangle these direct and indirect effects [1, 4]. This kind of information could be obtained from an increased global database about microbial communities considering the co-variables (more articles published), followed by meta-analysis and factorial experiments simultaneously testing pH and specific elements.

It could have important applications for agronomic and land reclamation systems. For example, there is a current research focus in identifying which bacterial species are related to plant yield, disease suppression, and environmental services [53, 54]. One possibility could be the stimulation of these bacteria using the knowledge about indirect effects of pH. Even though prokaryotic community structures and specific OTUs are affected by changes in the pH, the knowledge of specific indirect effects may allow a mineral supplementation to stimulate (or suppress) the growth of particular OTUs (e.g., Fe could be applied to stimulate *Bradyrhizobium* after a lime event).

Lastly, based on our conceptual model, we hypothesized that the pH effects on soil chemistry and in the microbial community structures could also affect microbial activity, such as gas fluxes [1, 31, 38]. We did not detect significant effects of the pH gradient on these fluxes at the sampling time but detected correlation with some microbial groups. It is likely, rather than investigating the relative abundance of specific OTUs, the quantification of marker genes related directly to the processes (e.g., *nosZ*, *mcrA*, and *pmoA*) simultaneously coupled to a temporal survey of the gases could be a better proxy to it, as demonstrated previously [31, 38].

## Conclusions

pH changes in soil co-occur with interactions among soil elements (e.g., precipitation of ions), hiding many indirect effects of pH on soil microbial communities. Thus, in this work, we investigated an important question that is rarely addressed in microbial ecology: is pH directly the main driver for most of the prokaryotic OTU occurrences or is it also a good indicator of the other co-variables that are in fact the relevant drivers for some microbial groups? Our data indicated that pH acts by means of two different mechanisms to drive prokaryotic community structures: (i) first, pH changes are directly associated with changes in some microbial groups, and (ii) the second is related to the “spillover effects” of pH, indirectly affecting microbial community structures through changes in some soil element availability. Moreover, indirect effects are highly relevant for some OTUs, and consequently for the community structure, deserving more attention in microbial ecology. Future studies in controlled conditions (e.g., factorial studies of pH and specific elements) should address this question in detail. Thereby, a better understanding of the ecology of several microbial groups can be more accurately accessed.

## Additional files

Additional file 1: Theoretical full diagram and rationally. **Figure S1A.** Overall theoretical diagram of expected interactions in the pH range between 4 and 6 with the microbial community structure. Boxes represent soil variables and the arrows the interactions (soluble Al^+ 3^ is toxic for plant roots and some bacteria; Fe, Mn, Cu, Zn, and B are nutrients that in high concentrations may be toxic; and P, K, Ca, Mg, and NO_3_ are nutrients rarely toxic to plants). In this study, the gradient was produced by liming application, with expected direct effect on pH, and Ca and Mg values. The indirect effects are the “spillover” effect of the pH in the other soil and plant variables. Indirect effect 1 is mainly related to the solubility of elements, while indirect effect 2 is related to these effects on plant growth and nutrient cycling. SOM is the abbreviated form for soil organic matter, CEC for cation exchange capacity, and WC for water content. Temperature and WC are considered in this diagram only for the survey day of greenhouse fluxes (proxy for microbial activity). **Figure S1B.** Effect of pH in the relative availability of important ions related to soil fertility (Fe, Cu, Mn, Zn, Al, Mo, Cl, P, N, S, B). (PDF 526 kb)

Additional file 2: Land-use description and soil parameters. **Table SI2A.** Land-use history of the crop fields (all the experimental plots were managed identically, the only difference between the treatments was the lime doses that created the pH gradient). **Table SI2B.** Soil chemical parameters, and plant yield, and greenhouse gas fluxes according to different soil pH (CaCl_2_) ranges. **Table SI2C.** Soil chemical parameters, plant yield, and greenhouse gas fluxes according to the lime doses. (PDF 775 kb)

Additional file 3: Hierarchical modeling of species communities (HMSC). **Table SI3A.** Predictive performance of different HMSC models based on fivefold cross-validation. All models include community-level random effect at the sample level. Predictive performance is measured by Tjur (2009) *R*^2^ for the presence–absence model and correlation for the abundance model. The values presented are averages over the OTUs. **Table SI3B.** Variance partitioning of the full HMSC models. The values show average (over the OTUs) proportion of variance attributed to each of the predictors. **Figure SI3A.** HMSC-based estimates of species responses to the environmental covariates. Panel A shows the results for the presence–absence model and panel B for the abundance model. In both cases, the OTUs have been ordered by their phylogeny, as illustrated by the plots. Positive and negative responses are shown by red and blue entries, respectively, and based on posterior mean. The darker red and blue colors corresponding to cases with strong statistical support (posterior probability at least 95%), and the percentages of such OTUs are given on the bottom of the panel. **Figure SI3B.** HMSC-based estimates of species residual (after accounting for influences of covariates) associations. Panels A and C show the results for the presence–absence model and panels B and D for the abundance model. In panels A and B, the species have been ordered in a way that best shows clusters of associated OTUs, whereas in panels C and D, they have been ordered by the phylogeny (as illustrated in the plots). Positive and negative OTU pairs for which the residual association is positive, with at least 95% posterior probability, are shown by red and blue entries, respectively. **Table SI3C.** Responses of the OTUs (− 1, negative; 1, positive; and only significant effects are shown *P* < 0.05) according to the abundance model to pH, Ca, Mg, indirect effects 1 (PCA1a and PCA1b), and indirect effects 2 (PCA2a and PCA2b). (PDF 1310 kb)

Additional file 4: Table S6 Random Forests (RF) models for each OTU (no significant values are reported as "0"). (XLSX 660 kb)

Additional file 5: Bacteria and Archaea Phyla correlations. Table S4 A1 Bacteria Relative Frequency (overall abundance >0.5%). Table S4 A2 Bacteria Relative Frequency (overall abundance <0.5%). Table S4 B1 Archaea Relative Frequency (related to Bacteria %). Table S4 B2 Archaea Relative Frequency (Only Archaea). Table S4 C Pearson (r) and Spearman (p) correlation indexes between soil parameters and the relative abundance of archaeal and bacterial phyla (only significant values are shown: r or ? >0.4 or <-0.4 and P<0.05). (XLS 57 kb)

Additional file 6: OTUs at Genus level (frequency and correlations). Table S5 A Spearman (p) and Pearson (r) correlation indexes between the soil chemical parameters and the relative abundance of archaeal and bacterial OTUs (only significant values are shown: r or p >0.4 or <-0.4 and P<0.05) (XLSX 229 kb)

Additional file 7: Phylogenetic tree correspondent to the Figure 3 - HMSC models. (PDF 17 kb)
