# Supplementary material for: Direct and indirect effects of a pH gradient bring insights into the mechanisms driving prokaryotic community structures
Source: Microbiome. 2018 Jun 11;6:106. doi: 10.1186/s40168-018-0482-8 (PMC5996553; doi:10.1186/s40168-018-0482-8)
Supplement: Supplementary file 1 — Theoretical full diagram and rationally. Figure S1A. Overall theoretical diagram of expected interactions in the pH range between 4 and 6 with the microbial community structure. Boxes represent soil variables and the arrows the interactions (soluble Al+ 3 is toxic for plant roots and some bacteria; Fe, Mn, Cu, Zn, and B are nutrients that in high concentrations may be toxic; and P, K, Ca, Mg, and NO3 are nutrients rarely toxic to plants). In this study, the gradient was produced by liming application, with expected direct effect on pH, and Ca and Mg values. The indirect effects are the “spillover” effect of the pH in the other soil and plant variables. Indirect effect 1 is mainly related to the solubility of elements, while indirect effect 2 is related to these effects on plant growth and nutrient cycling. SOM is the abbreviated form for soil organic matter, CEC for cation exchange capacity, and WC for water content. Temperature and WC are considered in this diagram only for the survey day of greenhouse fluxes (proxy for microbial activity). Figure S1B. Effect of pH in the relative availability of important ions related to soil fertility (Fe, Cu, Mn, Zn, Al, Mo, Cl, P, N, S, B). (PDF 526 kb) [file 40168_2018_482_MOESM1_ESM.pdf]

Lammel et al.: Direct and indirect effects of a pH gradient bring insights into the mechanisms driving prokaryotic community structures in a sub-tropical soil

Supplementary Information 1 Theoretical full diagram and rationally

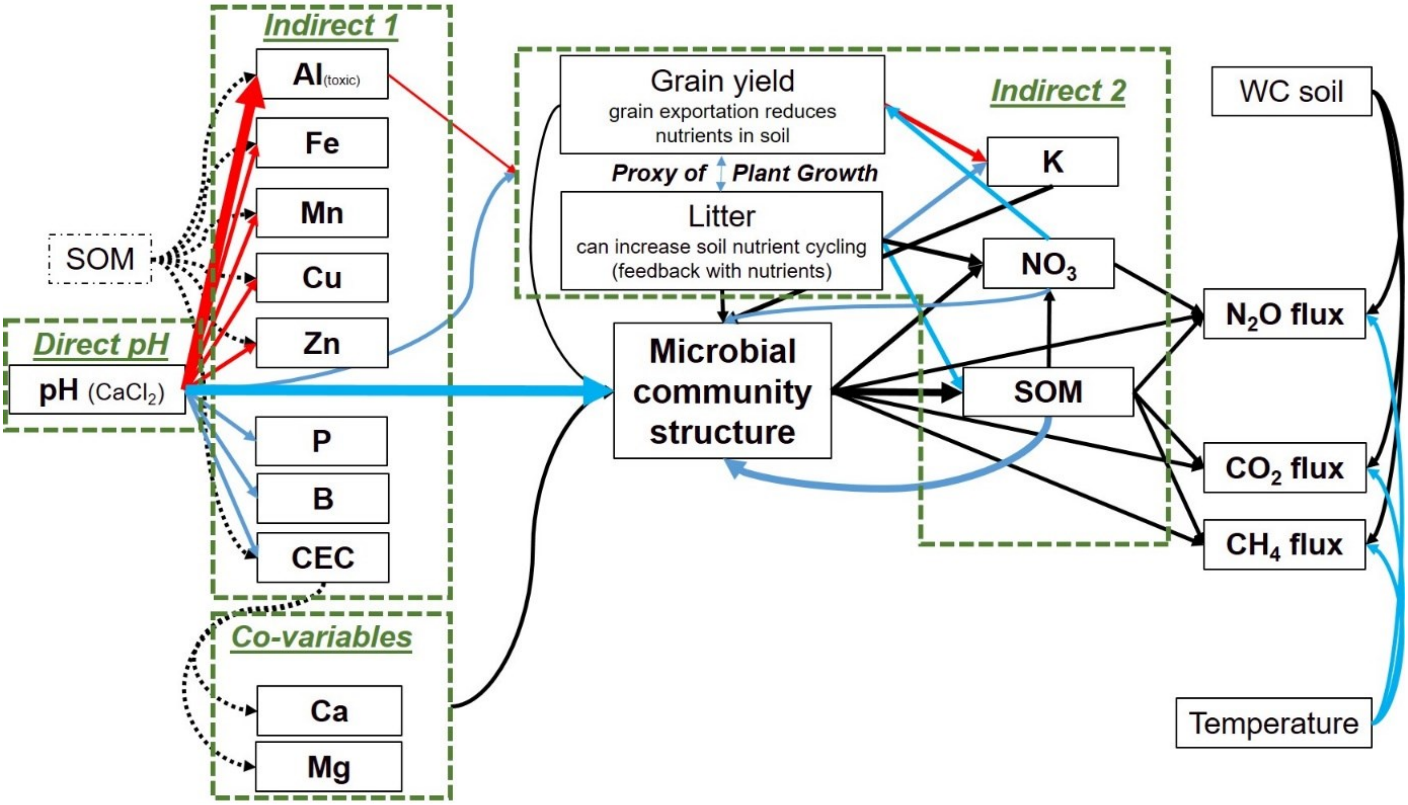

Figure S1-A Overall theoretical diagram of expected interactions in the pH range between 4-6 with the microbial community structure. Boxes represent soil variables and the arrows the interactions (soluble Al^{+3} is toxic for plant roots and some bacteria; Fe, Mn, Cu, Zn and B are nutrients that in high concentrations may be toxic; and P, K, Ca, Mg and NO_3 are nutrients rarely toxic to plants). In this study, the gradient was produced by liming application, with expected direct effect on pH, and Ca and Mg values. The indirect effects are the "spill-over" effect of the pH in the other soil and plant variables. Indirect effects 1 is mainly related to the solubility of elements, while indirect 2 is related to these effects on plant growth and nutrient cycling. SOM is abbreviation for soil organic matter, CEC, for cation exchange capacity, and WC, for water content. Temperature and WC are considered in this diagram only for the survey day of greenhouse fluxes (proxy for microbial activity).

Soil pH can have direct and indirect effects on soil microbiota (Figure S1-A). Direct effects are related to the proton concentration that affects microbial membranes, nutrient transporters and proteins stability and enzymatic activity [1-3]. While the indirect effects are related to nutrient availability (e.g. Fe, Cu, Mn, Zn, P, N, S and B), the availability of toxic elements (e.g. Al), and the effects on plant growth that drives organic inputs and chemical changes in soil [3-5].

The direct effects of pH are very like related to the H^+ potential that affect microbial membranes and nutrient transporters and metabolism; different prokaryotic species have different optimum pH and strategies to handle acidity [1, 3]. For example, acidophilic microorganisms usually have proteins with increased negative surface, and changes in the phospholipids membrane, at same time activing pumping H^+ out of the cells [2]. These organisms that are highly adapted for acidic conditions are likely to be outcompeted by other organisms better adapted to other pH ranges when pH changes (e.g. following liming) [6].

The indirect effects are rather complex. Soil pH is buffered by the soil colloids (clays and organic matter) and CEC. This also implies that a pH change in soil is expected to co-occur with a change in cation composition; usually the concentrations of Ca and Mg, the two most highly correlated co-variables with pH in soils worldwide [5]. Both elements are plant and microbial nutrients and are linked with several cellular and physiological processes [3, 5]. At the same time, increasing pH can precipitate cations, e.g. Al, Fe, Mn, Cu, Zn, thus reducing their biological availability (Figure S1-B). Consequently, these elements also act as buffers to soil pH changes [5].

We separated the indirect effects of pH into two main groups (Figure S1-A). The first group is related to the solubility of Al, Fe, Mn, Cu, Zn, P, B and to soil CEC (Figure

S1 B). Soluble Al^{+3} is known to be toxic for plants [7], and some studies previously reported potential negative impacts of Al in microbial communities [8-10]. Effects of Al toxicity in bacteria include Fe deficiency induction and metabolic disorders; however, overall effects on microbial cells are still largely unknown [11, 12].

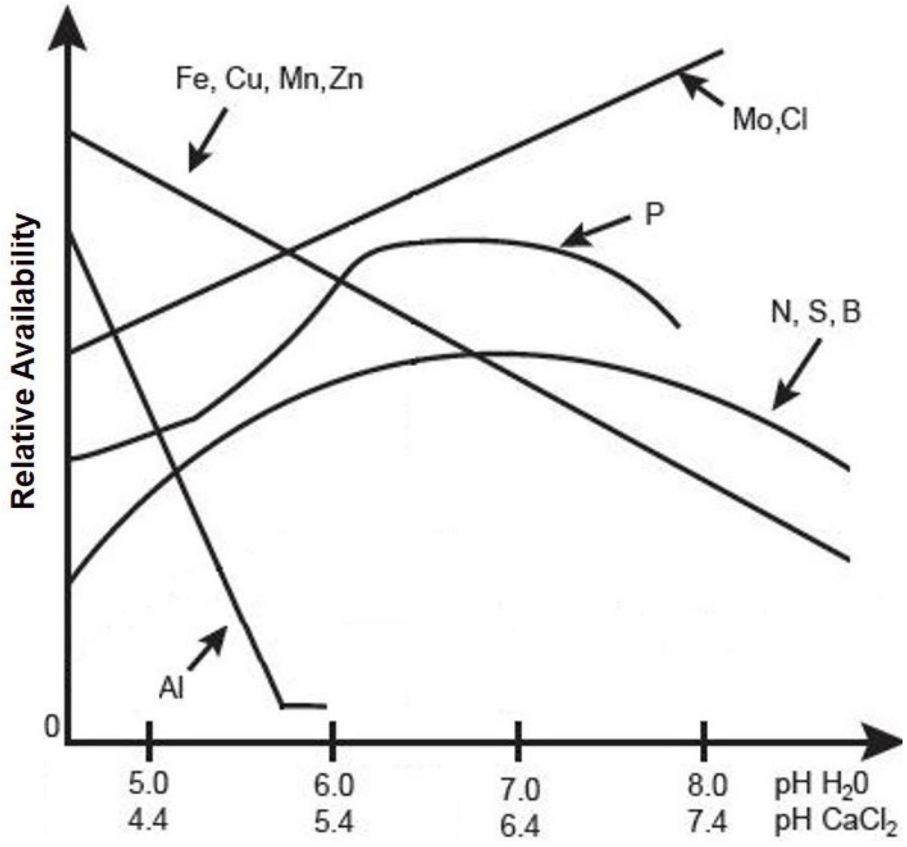

Figure S1-B Effect of pH in the relative availability of important ions related to soil fertility (Fe, Cu, Mn, Zn, Al, Mo, Cl, P, N, S, B). Adapted from Malavolta [13] and IPNI-Lopes [14].

Effects of Fe, Mn, Cu, Zn, B, and P were previously reported on bacterial studies [10, 15, 16]. Even though these elements are nutrients when available in optimal concentrations, they can be toxic (at variable thresholds for different microbial groups), and different microbial groups also have different strategies for their uptake and detoxification [3, 12, 17]. The role of iron in soil microbial communities is complex,

involving interactions with soil and plants [4, 18]. Mn, Cu, Zn and B are important micronutrient linked to enzymatic activity, but in excess Mn can inhibit enzymatic activity [19], Cu can be toxic [20], Zn can cause metabolic disorder [21], and B may impair protein synthesis in microorganisms [22]. P is an important microbial nutrient and its role is widely studied in soils [23]. The last factor in indirect effects 1 is soil CEC, that is the result of the colloid (clays) surface charges, and thus may interact with microbial cells and biofilm formation, and can influence cation availability [3, 24].

The second group of indirect effects is related to plant growth and other soil parameters. Due to all the above mentioned factors, pH can change plant biomass accumulation and grain production, and thus affect the nutrient dynamic in soils. Plants can compete for nutrients with microbial communities [25], and nutrients from different depths in soils can be exported by the harvest or cycled to the soil upper layers through plant litter [26]. Plant litter accumulation on the soil surface can especially increase SOM, K and N in the top soil, and these are also known drivers for microbial community structures [26, 27]. Moreover, plants that grow in very acidic soils (pH<5.5) may produce organic exudates to reduce the toxicity of Al^{+3}, and after changing soil pH, plant exudates and litter composition can change and may also influence the microbial community [4, 7, 28, 29].

1. Slonczewski JL, Fujisawa M, Dopson M, Krulwich TA. Cytoplasmic pH Measurement and Homeostasis in Bacteria and Archaea. Adv Microb Physiol. 2009;55:1-79.
2. Krulwich TA, Sachs G, Padan E. Molecular aspects of bacterial pH sensing and homeostasis. Nat Rev Microbiol. 2011;9:330-343.
3. Madigan MT. Brock biology of microorganisms, Fourteenth edition. edn. Boston: Pearson; 2015.
4. Rengel Z. Availability of Mn, Zn and Fe in the rhizosphere. J Soil Sci Plant Nut. 2015;15:397-409.
5. Weil. Ray R. Brady NC, Weil RR. The nature and properties of soils, Fifteenth edition. edn. Columbus: Pearson; 2016.
6. Rousk J, Baath E, Brookes PC, Lauber CL, Lozupone C, Caporaso JG, Knight R, Fierer N. Soil bacterial and fungal communities across a pH gradient in an arable soil. Isme J. 2010;4:1340-1351.

7. Kochian LV, Pineros MA, Liu JP, Magalhaes JV. Plant Adaptation to Acid Soils: The Molecular Basis for Crop Aluminum Resistance. Annu Rev Plant Biol. 2015;66:571-598.
8. Ozawa T, Imai Y, Sukiman HI, Karsono H, Ariani D, Saono S. Low pH and aluminum tolerance of Bradyrhizobium strains isolated from acid soils in Indonesia. Soil Sci Plant Nutr. 1999;45:987-992.
9. Faoro H, Alves AC, Souza EM, Rigo LU, Cruz LM, Al-Janabi SM, Monteiro RA, Baura VA, Pedrosa FO. Influence of Soil Characteristics on the Diversity of Bacteria in the Southern Brazilian Atlantic Forest. Appl Environ Microb. 2010;76:4744-4749.
10. Mendes LW, Brossi MJD, Kuramae EE, Tsai SM. Land-use system shapes soil bacterial communities in Southeastern Amazon region. Appl Soil Ecol. 2015;95:151-160.
11. Lemire J, Mailloux R, Auger C, Whalen D, Appanna VD. Pseudomonas fluorescens orchestrates a fine metabolic-balancing act to counter aluminium toxicity. Environ Microbiol. 2010;12:1384-1390.
12. Lemire JA, Harrison JJ, Turner RJ. Antimicrobial activity of metals: mechanisms, molecular targets and applications. Nat Rev Microbiol. 2013;11:371-384.
13. Malavolta E. Manual de química agrícola : adubos e adubação., 2nd edn. São Paulo-SP: Agronômica Ceres; 1967.
14. IPNI-Lopes. Manual Internacional de Fertilidade do Solo, 2 ed. edn. Piracicaba: International Plant Nutrition Institute (IPNI); 1998.
15. Zhalnina K, Dias R, de Quadros PD, Davis-Richardson A, Camargo FAO, Clark IM, McGrath SP, Hirsch PR, Triplett EW. Soil pH Determines Microbial Diversity and Composition in the Park Grass Experiment. Microb Ecol. 2015;69:395-406.
16. Navarrete AA, Mellis EV, Escalas A, Lemos LN, Lavres J, Quaggio JA, Zhou JZ, Tsai SM. Zinc concentration affects the functional groups of microbial communities in sugarcane-cultivated soil. Agr Ecosyst Environ. 2017;236:187-197.
17. Giller KE, Witter E, McGrath SP. Heavy metals and soil microbes. Soil Biol Biochem. 2009;41:2031-2037.
18. Marschner P, Crowley D, Rengel Z. Rhizosphere interactions between microorganisms and plants govern iron and phosphorus acquisition along the root axis - model and research methods. Soil Biol Biochem. 2011;43:883-894.
19. Hohle TH, O'Brian MR. Magnesium-dependent processes are targets of bacterial manganese toxicity. Mol Microbiol. 2014;93:736-747.
20. Ladomersky E, Petris MJ. Copper tolerance and virulence in bacteria. Metallomics. 2015;7:957-964.
21. Hantke K. Bacterial zinc uptake and regulators. Curr Opin Microbiol. 2005;8:196-202.
22. Sayin Z, Ucan US, Sakmanoglu A. Antibacterial and Antibiofilm Effects of Boron on Different Bacteria. Biol Trace Elem Res. 2016;173:241-246.
23. Capek P, Kotas P, Manzoni S, Santruckova H. Drivers of phosphorus limitation across soil microbial communities. Funct Ecol. 2016;30:1705-1713.
24. Stotzky G. Influence of clay minerals on microorganisms: III. Effect of particle size, cation exchange capacity, and surface area on bacteria. Can J Microbiol. 1966;12:1235–1246.
25. Zhu Q, Riley WJ, Tang J, Koven CD. Multiple soil nutrient competition between plants, microbes, and mineral surfaces: model development, parameterization, and example applications in several tropical forests. Biogeosciences. 2016;13:341-363.
26. Zhang GQ, Zhang P, Peng SZ, Chen YM, Cao Y. The coupling of leaf, litter, and soil nutrients in warm temperate forests in northwestern China. Sci Rep-Uk. 2017;7.
27. Lammel DR, Nusslein K, Tsai SM, Cerri CC. Land use, soil and litter chemistry drive bacterial community structures in samples of the rainforest and Cerrado (Brazilian Savannah) biomes in Southern Amazonia. Eur J Soil Biol. 2015;66:32-39.
28. Eisenhauer N, Lanoue A, Strecker T, Scheu S, Steinauer K, Thakur MP, Mommer L. Root biomass and exudates link plant diversity with soil bacterial and fungal biomass. Sci Rep-Uk. 2017;7.
29. Young E, Carey M, Meharg AA, Meharg C. Microbiome and ecotypic adaption of Holcus lanatus (L.) to extremes of its soil pH range, investigated through transcriptome sequencing. Microbiome. 2018;6.
