# Supplementary material for: Direct and indirect effects of a pH gradient bring insights into the mechanisms driving prokaryotic community structures
Source: Microbiome. 2018 Jun 11;6:106. doi: 10.1186/s40168-018-0482-8 (PMC5996553; doi:10.1186/s40168-018-0482-8)
Supplement: Supplementary file 2 — Land-use description and soil parameters. Table SI2A. Land-use history of the crop fields (all the experimental plots were managed identically, the only difference between the treatments was the lime doses that created the pH gradient). Table SI2B. Soil chemical parameters, and plant yield, and greenhouse gas fluxes according to different soil pH (CaCl2) ranges. Table SI2C. Soil chemical parameters, plant yield, and greenhouse gas fluxes according to the lime doses. (PDF 775 kb) [file 40168_2018_482_MOESM2_ESM.pdf]

*Lammel et al.: Direct and indirect effects of a pH gradient bring insights into the mechanisms driving prokaryotic community structures in a sub-tropical soil*

**Supplementary Information 2** Land use description and soil parameters

**Table SI2 A** Land use history of the crop fields (all the experimental plots were managed identically, the only difference between the treatments was the lime doses - that created the pH gradient)

| | | | Furrow fertilization | | Broadcast fertilization | |
|---|---|---|---|---|---|---|
| **Year** | **Crop** | **Season** | **Source ($N$-$P_2O_5$-$K_2O$)** | **Dose (kg ha$^{-1}$)** | **Source** | **Dose (kg ha$^{-1}$)** |
| 2010 | Black Oat | Winter | 12-52-00 | 200 | - | - |
| 2010/11 | Corn | Spring/Summer | 14-34-00 | 350 | 22-00-21 and Urea | 225 and 435 |
| 2011 | Black Oat | Winter | 14-34-00 | 313 | - | - |
| 2011/12 | Soybean | Spring/Summer | - | - | KCl | 125 |
| 2012 | Black Oat | Winter | 19-38-00 | 260 | KCl | 120 |
| 2012/13 | Soybean | Spring/Summer | - | - | - | - |
| 2013 | Black Oat | Winter | 14-34-00 | 337 | KCl | 170 |
| 2013/14 | Soybean | Spring/Summer | - | - | - | - |

**Table SI2 B** Soil chemical parameters, and plant yield, and greenhouse gas fluxes according to different soil pH ($CaCl_2$) ranges

| Parameter | (unit) | pH range 4.0-4.4 | pH range 4.5-5.0 | pH range 5.1-5.5 | pH range 5.6-6.2 |
|---|---|---|---|---|---|
| *Soil Chemistry* | | | | | |
| **pH ($CaCl_2$)** | | 4.1 ± *0.1* d[1] | 4.7 ± *0.2* c | 5.2 ± *0.1* b | 6.1 ± *0.1* a |
| **pH ($H_2O$)** | | 5.2 ± *0.1* d | 5.6 ± *0.2* c | 6.0 ± *0.1* b | 6.7 ± *0.1* a |
| **H+Al** | $cmol_c.dm^3$ | 97.6 ± *14.4* a | 57.3 ± *11.0* b | 39.0 ± *4.1* c | 21.0 ± *1.4* d |
| **Al** | $cmol_c.dm^3$ | 13.1 ± *3.1* a | 2.1 ± *1.5* b | 0.1 ± *0.1* c | 0.0 ± *0.0* c |
| **K** | $cmol_c.dm^3$ | 4.4 ± *0.5* a | 4.0 ± *0.9* a | 4.5 ± *0.3* a | 3.9 ± *0.5* a |
| **Ca** | $cmol_c.dm^3$ | 19.1 ± *2.5* d | 48.3 ± *14.4* c | 74.1 ± *18.4* b | 128.5 ± *14.8* a |
| **Mg** | $cmol_c.dm^3$ | 6.8 ± *1.2* b | 16.1 ± *7.6* a | 24.4 ± *17.8* ab | 15.5 ± *4.9* a |
| **CEC** | $cmol_c.dm^3$ | 128 ± *15* b | 126 ± *10* b | 142 ± *27* ab | 169 ± *21* a |
| **P** | $mg.dm^3$ | 38.8 ± *6.0* b | 52.5 ± *13.0* a | 44.6 ± *8.4* ab | 49.0 ± *25.5* ab |
| **B** | $mg.dm^3$ | 0.45 ± *0.08* a | 0.39 ± *0.05* ab | 0.38 ± *0.05* ab | 0.31 ± *0.06* b |
| **Fe** | $mg.dm^3$ | 89 ± *36* a | 70 ± *20* ab | 59 ± *16* b | 39 ± *0* b |
| **Mn** | $mg.dm^3$ | 4.0 ± *0.6* a | 3.5 ± *0.8* ab | 3.3 ± *0.5* b | 3.5 ± *0.4* ab |
| **Cu** | $mg.dm^3$ | 1.6 ± *0.3* a | 1.4 ± *0.2* ab | 1.4 ± *0.2* ab | 1.2 ± *0.0* b |
| **Zn** | $mg.dm^3$ | 0.60 ± *0.34* a | 0.45 ± *0.09* a | 0.59 ± *0.23* a | 0.45 ± *0.07* a |
| **SOM** | $g.dm^3$ | 55 ± *7* a | 50 ± *7* a | 52 ± *4* a | 46 ± *3* a |
| **$NO_3$** | $mg.kg^{-1}$ | 6.6 ± *0.8* a | 6.2 ± *1.4* a | 7.1 ± *0.4* a | 5.6 ± *0.3* a |
| *Plant Yield* | | | | | |
| **Soy Yield** | $kg.ha^{-1}$ | 4104 ± *311* b | 4334 ± *293* ab | 4425 ± *538* ab | 4717 ± *19* a |
| **Litter** | $g.m^2$ | 85 ± *22* a | 88 ± *44* a | 96 ± *47* a | 77 ± *41* a |
| *Greenhouse gas* | | | | | |
| **$CO_2$ flux** | $mg.m^2.h^{-1}$ | 65 ± *57* a | 122 ± *110* a | 130 ± *75* a | 104 ± *51* a |
| **$CH_4$ flux** | $ug.m^2.h^{-1}$ | -8 ± *14* a | -4 ± *22* a | -8 ± *21* a | -24 ± *4* a |
| **$N_2O$ flux** | $ug.m^2.h^{-1}$ | 4 ± *49* a | 25 ± *63* a | -7 ± *64* a | 74 ± *62* a |

[1] Values with the same letter in one row are not different by Tukey’s post-hoc test ($p<0.05$).

**Table SI2 C** Soil chemical parameters, plant yield, and greenhouse gas fluxes according to the lime doses

| Parameter | (unit) | Lime Dose (kg.ha$^{-1}$) | | | |
|---|---|---|---|---|---|
| | | 0 | 2250 | 4500 | 6750 |
| *Soil Chemistry* | | | | | |
| **pH ($CaCl_2$)** | | 4.2 ± 0.3 | 4.6 ± 0.2 | 5.0 ± 0.2 | 5.3 ± 0.5 |
| **pH ($H_2O$)** | | 5.3 ± 0.3 | 5.5 ± 0.1 | 5.8 ± 0.1 | 6.1 ± 0.4 |
| **H+Al** | $cmol_c.dm^3$ | 79 ± 22 | 62 ± 8 | 45 ± 5 | 38 ± 12 |
| **Al** | $cmol_c.dm^3$ | 11.7 ± 4.9 | 3.1 ± 1.3 | 0.5 ± 0.3 | 0.5 ± 0.8 |
| **K** | $cmol_c.dm^3$ | 4.4 ± 0.4 | 4.1 ± 0.9 | 4.3 ± 0.7 | 4.0 ± 0.7 |
| **Ca** | $cmol_c.dm^3$ | 22.9 ± 10.9 | 42.8 ± 13.7 | 62.0 ± 11.5 | 81.4 ± 31.8 |
| **Mg** | $cmol_c.dm^3$ | 9.6 ± 8.0 | 11.9 ± 2.4 | 20.7 ± 8.6 | 21.3 ± 14.8 |
| **CEC** | $cmol_c.dm^3$ | 128 ± 13 | 123 ± 9 | 133 ± 8 | 145 ± 28 |
| **P** | $mg.dm^3$ | 38.9 ± 5.3 | 50.9 ± 13.8 | 51.7 ± 14.7 | 48.9 ± 9.3 |
| **B** | $mg.dm^3$ | 0.4 ± 0.1 | 0.4 ± 0.0 | 0.4 ± 0.0 | 0.4 ± 0.1 |
| **Fe** | $mg.dm^3$ | 85 ± 34 | 74 ± 21 | 59 ± 9 | 61 ± 21 |
| **Mn** | $mg.dm^3$ | 3.9 ± 0.6 | 3.8 ± 0.5 | 3.4 ± 0.3 | 3.1 ± 0.7 |
| **Cu** | $mg.dm^3$ | 1.6 ± 0.2 | 1.5 ± 0.2 | 1.5 ± 0.2 | 1.3 ± 0.2 |
| **Zn** | $mg.dm^3$ | 0.6 ± 0.3 | 0.5 ± 0.1 | 0.5 ± 0.1 | 0.5 ± 0.2 |
| **SOM** | $g.dm^3$ | 54 ± 7 | 49 ± 5 | 55 ± 3 | 48 ± 7 |
| **$NO_3$** | $mg.kg^{-1}$ | 6.7 ± 0.7 | 6.6 ± 1.3 | 5.9 ± 1.5 | 6.5 ± 0.8 |
| *Plant Yield* | | | | | |
| **Soy Yield** | $kg.ha^{-1}$ | 4061 ± 300 | 4206 ± 189 | 4578 ± 156 | 4510 ± 436 |
| **Litter** | $g.m^2$ | 79 ± 18 | 94 ± 44 | 66 ± 26 | 81 ± 39 |
| *Gas fluxes* | | | | | |
| **$CO_2$ flux** | $mg.m^2.h^{-1}$ | 63 ± 51 | 112 ± 81 | 94 ± 61 | 153 ± 114 |
| **$CH_4$ flux** | $ug.m^2.h^{-1}$ | -12 ± 17 | 0 ± 27 | -2 ± 7 | -10 ± 15 |
| **$N_2O$ flux** | $ug.m^2.h^{-1}$ | 1 ± 44 | 37 ± 70 | -16 ± 43 | 24 ± 62 |
| **WC (soil)** | **(%)** | 26.6 ± 1.1 | 26.7 ± 1.0 | 26.3 ± 1.1 | 26.5 ± 0.8 |
| **Temperature** | (C) | 25.8 ± 2.3 | 24.7 ± 3.4 | 25.4 ± 2.7 | 26.3 ± 2.6 |
