# Supplementary material for: Direct and indirect effects of a pH gradient bring insights into the mechanisms driving prokaryotic community structures
Source: Microbiome. 2018 Jun 11;6:106. doi: 10.1186/s40168-018-0482-8 (PMC5996553; doi:10.1186/s40168-018-0482-8)
Supplement: Supplementary file 3 — Hierarchical modeling of species communities (HMSC). Table SI3A. Predictive performance of different HMSC models based on fivefold cross-validation. All models include community-level random effect at the sample level. Predictive performance is measured by Tjur (2009) R2 for the presence–absence model and correlation for the abundance model. The values presented are averages over the OTUs. Table SI3B. Variance partitioning of the full HMSC models. The values show average (over the OTUs) proportion of variance attributed to each of the predictors. Figure SI3A. HMSC-based estimates of species responses to the environmental covariates. Panel A shows the results for the presence–absence model and panel B for the abundance model. In both cases, the OTUs have been ordered by their phylogeny, as illustrated by the plots. Positive and negative responses are shown by red and blue entries, respectively, and based on posterior mean. The darker red and blue colors corresponding to cases with strong statistical support (posterior probability at least 95%), and the percentages of such OTUs are given on the bottom of the panel. Figure SI3B. HMSC-based estimates of species residual (after accounting for influences of covariates) associations. Panels A and C show the results for the presence–absence model and panels B and D for the abundance model. In panels A and B, the species have been ordered in a way that best shows clusters of associated OTUs, whereas in panels C and D, they have been ordered by the phylogeny (as illustrated in the plots). Positive and negative OTU pairs for which the residual association is positive, with at least 95% posterior probability, are shown by red and blue entries, respectively. Table SI3C. Responses of the OTUs (− 1, negative; 1, positive; and only significant effects are shown P < 0.05) according to the abundance model to pH, Ca, Mg, indirect effects 1 (PCA1a and PCA1b), and indirect effects 2 (PCA2a and PCA2b). (PDF 1310 kb) [file 40168_2018_482_MOESM3_ESM.pdf]

Supplementary Information 3 Hierarchical Modelling of Species Communities (HMSC)

Table SI3 A Predictive performance of different HMSC models based on five-fold cross-validation. All models include community-level random effect at the sample level. Predictive performance is measured by Tjur (2009) R^2 for the presence-absence model and correlation for the abundance model. The values presented are averages over the OTUs

Included predictors	Presence-absence model	Abundance model
G1	0.05	0.22
G1+G2	0.05	0.18
G1+G2+G3	0.05	0.28
G1+G2+G4	0.06	0.23
G1+G2+G3+G4	0.06	0.29

Table SI3 B Variance partitioning of the full HMSC models. The values show average (over the OTUs) proportion of variance attributed to each of the predictors

Predictor	Presence-absence model	Abundance model
G1	33%	33%
G2	19%	6%
G3	18%	18%
G4	17%	10%
random effect	12%	33%

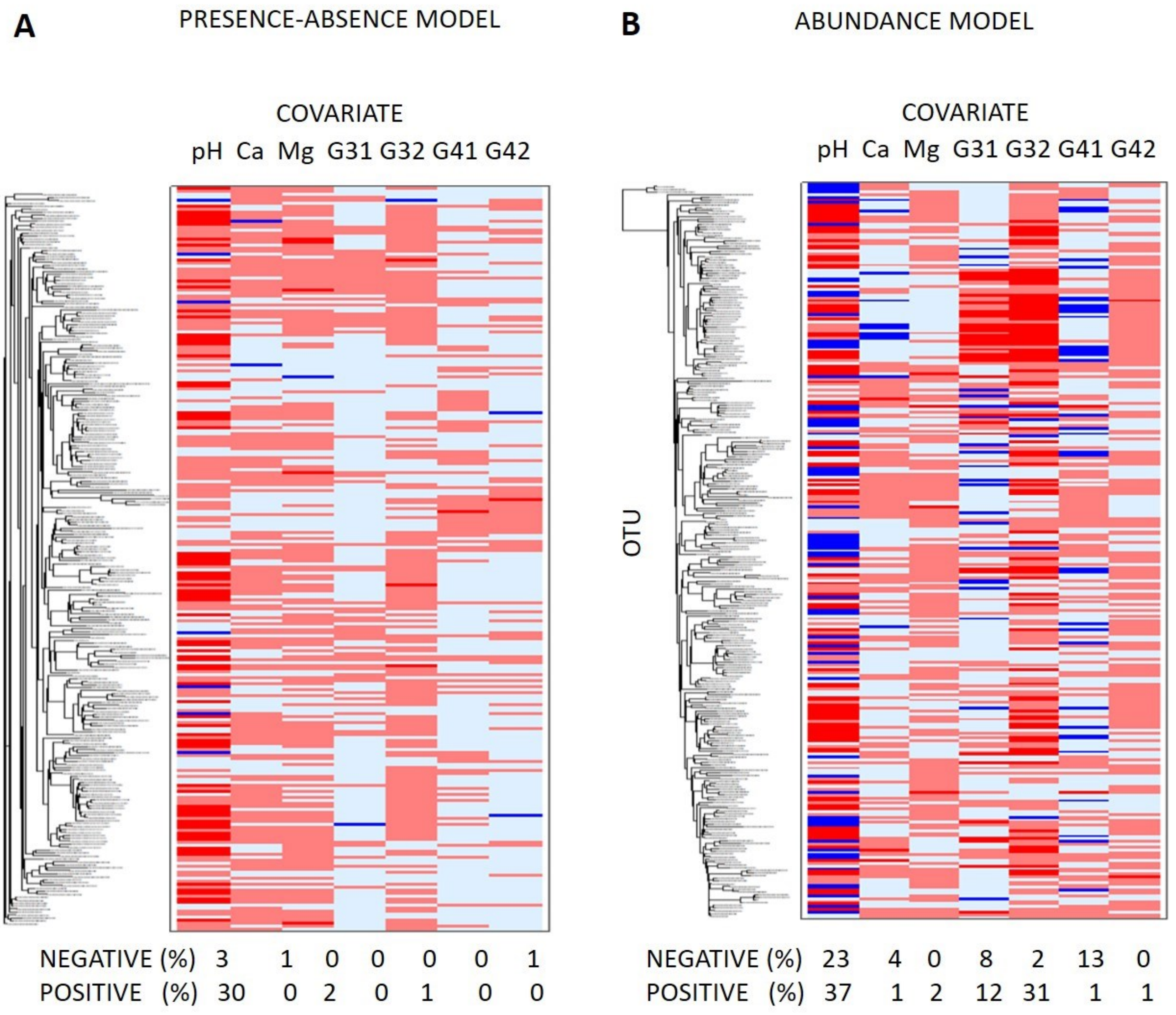

Fig. SI3 A HMSC-based estimates of species responses to the environmental covariates. **Panel A** shows the results for the presence-absence model and **panel B** for the abundance model. In both cases, the OTUs have been ordered by their phylogeny, as illustrated by the plots. Positive or negative responses are shown by red and blue entries, respectively and based on posterior mean. The darker red and blue colors corresponding to cases with strong statistical support (posterior probability at least 95%), and the percentages of such OTUs are given on the bottom of the panel

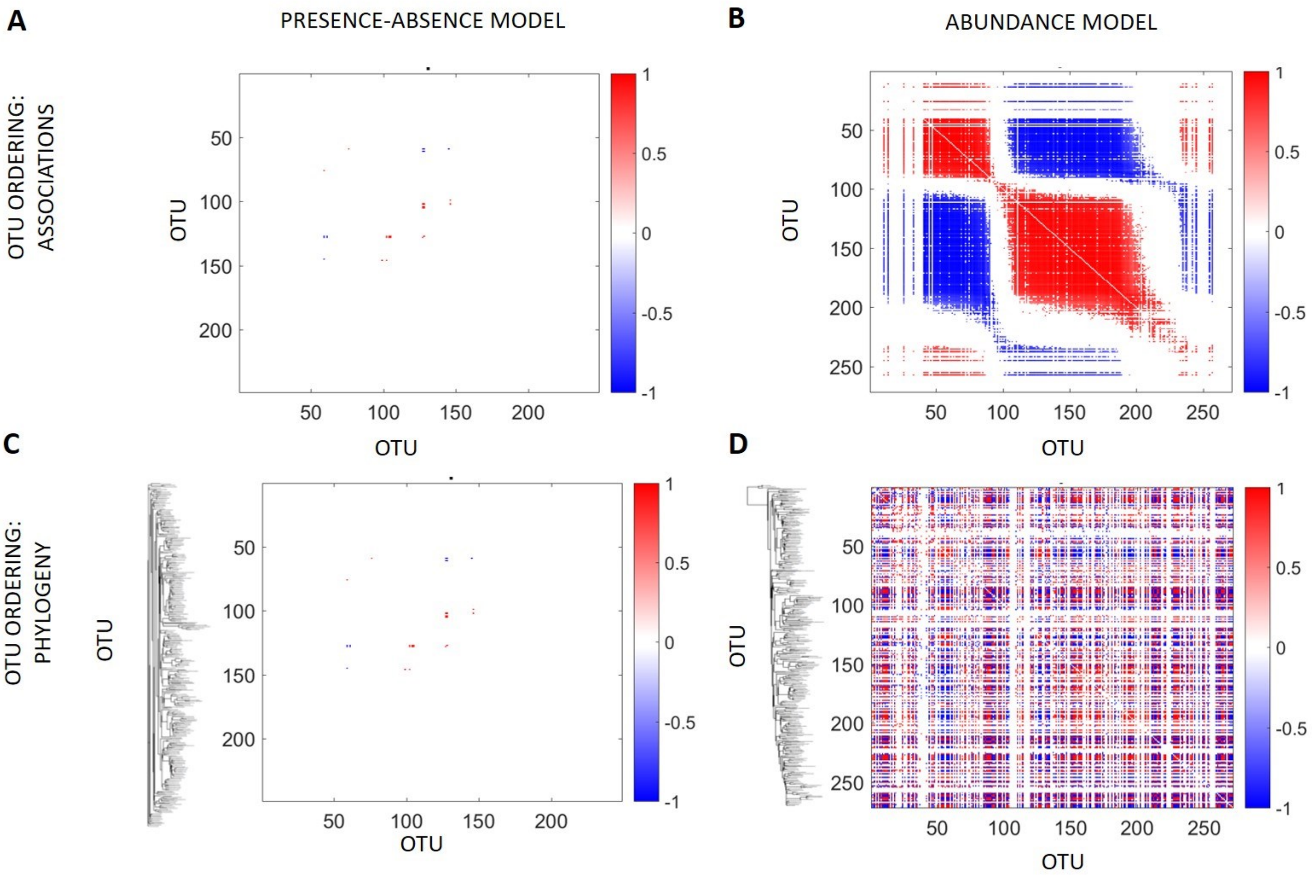

Fig. SI3 B HMSC-based estimates of species residual (after accounting for influences of covariates) associations. **Panels AC** show the results for the presence-absence model and **panels BD** for the abundance model. In panels AB, the species have been ordered in a way that best shows clusters of associated OTUs, whereas in panels CD they have been ordered by the phylogeny (as illustrated in the plots). Positive and negative OTU pairs for which the residual association is positive, with at least 95% posterior probability, is shown by red and blue entries, respectively

Table SI3 C Responses of the OTUs (-1, negative; 1, positive; only significant effects are shown *P*<0.05) according to the abundance model to pH, Ca, Mg, Indirect Effects 1 (PCA1a and PCA1b) and Indirect Effects 2 (PCA2a and PCA2b)

OTU	pH	Ca	Mg	PCA1a	PCA1b	PCA2a	PCA2b
OTU1112_p_Proteobacteria_g_Anaeromyxobacter	-1	-1		1			
OTU1318_p_TM6_Ambiguous_taxa	-1	1			1		
OTU1104_p_Proteobacteria_	-1		1	-1	-1	1	
OTU1329_p_Verrucomicrobia_g_uncultured_bacterium	-1		1			1	
OTU1155_p_Proteobacteria_g_uncultured_bacterium	-1		1				
OTU1101_p_Proteobacteria_	-1			-1	-1		
OTU1119_p_Proteobacteria_g_uncultured_bacterium	-1			1	1		
OTU1120_p_Proteobacteria_	-1			1	1		
OTU1121_p_Proteobacteria_g_uncultured_bacterium	-1			1	1		
OTU1131_p_Proteobacteria_g_uncultured_bacterium	-1			1	1		
OTU1137_p_Proteobacteria_g_Byssovorax	-1			1	1		
OTU1278_p_Proteobacteria_g_Rhodanobacter	-1			1		-1	
OTU1288_p_Proteobacteria_g_Steroidobacter	-1			1		-1	
OTU1215_p_Proteobacteria_g_uncultured	-1			1			
OTU1271_p_Proteobacteria_g_Dokdonella	-1			1			
OTU1287_p_Proteobacteria_g_Acidibacter	-1			1			
OTU1274_p_Proteobacteria_g_Lysobacter	-1				-1		
OTU1331_p_Verrucomicrobia_g_uncultured	-1				-1		
OTU1134_p_Proteobacteria_g_Phaselicystis	-1				1		
OTU1139_p_Proteobacteria_g_Sorangium	-1				1		
OTU1129_p_Proteobacteria_	-1					-1	
OTU1065_p_Proteobacteria_	-1						
OTU1066_p_Proteobacteria_Ambiguous_taxa	-1						
OTU1067_p_Proteobacteria_g_uncultured_bacterium	-1						
OTU1079_p_Proteobacteria_g_Bdellovibrio	-1						
OTU1087_p_Proteobacteria_g_Candidatus_Entotheonella	-1						
OTU1090_p_Proteobacteria_	-1						
OTU1096_p_Proteobacteria_	-1						
OTU1097_p_Proteobacteria_Ambiguous_taxa	-1						
OTU1098_p_Proteobacteria_g_uncultured_bacterium	-1						
OTU1108_p_Proteobacteria_g_uncultured_bacterium	-1						
OTU1110_p_Proteobacteria_g_uncultured_bacterium	-1						
OTU1117_p_Proteobacteria_g_Haliangium	-1						
OTU1143_p_Proteobacteria_g_uncultured	-1						
OTU1148_p_Proteobacteria_g_uncultured_bacterium	-1						
OTU1152_p_Proteobacteria_	-1						
OTU1157_p_Proteobacteria_g_uncultured_bacterium	-1						
OTU1163_p_Proteobacteria_g_uncultured_bacterium	-1						

(*continuation*)

OTU	pH	Ca	Mg	PCA1a	PCA1b	PCA2a	PCA2b
OTU1211_p_Proteobacteria_g_Aquicella	-1						
OTU1212_p_Proteobacteria_g_Coxiella	-1						
OTU1217_p_Proteobacteria_g_Legionella	-1						
OTU1222_p_Proteobacteria_g_uncultured_bacterium	-1						
OTU1262_p_Proteobacteria_	-1						
OTU1264_p_Proteobacteria_g_uncultured_bacterium	-1						
OTU1284_p_Proteobacteria_g_uncultured	-1						
OTU1289_p_Proteobacteria_g_uncultured	-1						
OTU1316_p_Thermotogae_g_GAL15	-1						
OTU1319_p_TM6_g_uncultured_bacterium	-1						
OTU1327_p_Verrucomicrobia_	-1						
OTU1328_p_Verrucomicrobia_Ambiguous_taxa	-1						
OTU1333_p_Verrucomicrobia_g_Pedosphaera	-1						
OTU1336_p_Verrucomicrobia_g_Opitutus	-1						
OTU1341_p_Verrucomicrobia_g_Chthoniobacter	-1						
OTU1342_p_Verrucomicrobia_	-1						
OTU1344_p_Verrucomicrobia_g_uncultured_bacterium	-1						
OTU1345_p_Verrucomicrobia_g_uncultured_Spartobacteria	-1						
OTU1353_p_Verrucomicrobia_g_Candidatus_Xiphinematobacter	-1						
OTU1369_p_WCHB1_60_g_uncultured_bacterium	-1						
OTU1371_p_WD272_	-1						
OTU1372_p_WD272_Ambiguous_taxa	-1						
OTU1373_p_WD272_g_uncultured_bacterium	-1						
OTU1374_Unassigned	-1						
OTU75_p_Acidobacteria_Ambiguous_taxa	1	-1		1	1		
OTU83_p_Acidobacteria_	1	-1			1	-1	1
OTU67_p_Acidobacteria_Ambiguous_taxa	1	-1			1		
OTU110_p_Actinobacteria_	1	-1				-1	
OTU114_p_Actinobacteria_g_uncultured	1	-1				-1	
OTU215_p_Actinobacteria_g_Kribbella	1	1					
OTU23_Bacteria_unknown	1		1				
OTU195_p_Actinobacteria_g_Actinoplanes	1			-1	1	-1	
OTU128_p_Actinobacteria_g_Mycobacterium	1			-1	1		
OTU133_p_Actinobacteria_g_Rhodococcus	1			-1	1		
OTU300_p_Armatimonadetes_g_uncultured_bacterium	1			-1		-1	
OTU87_p_Acidobacteria_g_uncultured_bacterium	1			-1			
OTU123_p_Actinobacteria_g_uncultured_bacterium	1			-1			
OTU175_p_Actinobacteria_g_Terrabacter	1			-1			
OTU199_p_Actinobacteria_g_Hamadaea	1			-1			
OTU275_p_Actinobacteria_g_uncultured_actinobacterium	1			-1			
OTU277_p_Actinobacteria_g_Conexibacter	1			-1			
OTU408_p_Chlamydiae_g_Neochlamydia	1			-1			
OTU79_p_Acidobacteria_	1			1	1	-1	

(*continuation*)

OTU	pH	Ca	Mg	PCA1a	PCA1b	PCA2a	PCA2b
OTU80_p_Acidobacteria_Ambiguous_taxa	1			1	1	-1	
OTU78_p_Acidobacteria_g_Blastocatella	1			1	1		
OTU201_p_Actinobacteria_g_Luedemannella	1			1	1		
OTU65_p_Acidobacteria_g_Candidatus_Solibacter	1				1	-1	
OTU81_p_Acidobacteria_g_uncultured_Acidobacteria	1				1	-1	
OTU82_p_Acidobacteria_g_uncultured_bacterium	1				1	-1	
OTU193_p_Actinobacteria_Ambiguous_taxa	1				1	-1	
OTU216_p_Actinobacteria_g_Marmoricola	1				1	-1	
OTU217_p_Actinobacteria_g_Nocardioides	1				1	-1	
OTU261_p_Actinobacteria_g_Gaiella	1				1	-1	
OTU356_p_Bacteroidetes_g_Niastella	1				1	-1	
OTU10_p_Thaumarchaeota_Ambiguous_taxa	1				1		
OTU26_p_Acidobacteria_	1				1		
OTU31_p_Acidobacteria_g_Edaphobacter	1				1		
OTU35_p_Acidobacteria_g_uncultured	1				1		
OTU40_p_Acidobacteria_	1				1		
OTU51_p_Acidobacteria_Ambiguous_taxa	1				1		
OTU53_p_Acidobacteria_g_uncultured_bacterium	1				1		
OTU64_p_Acidobacteria_g_Bryobacter	1				1		
OTU69_p_Acidobacteria_g_uncultured_bacterium	1				1		
OTU85_p_Acidobacteria_g_uncultured_Acidobacteria	1				1		
OTU86_p_Acidobacteria_g_uncultured_Acidobacteriales	1				1		
OTU96_p_Acidobacteria_g_uncultured_bacterium	1				1		
OTU119_p_Actinobacteria_	1				1		
OTU120_p_Actinobacteria_Ambiguous_taxa	1				1		
OTU132_p_Actinobacteria_g_Nocardia	1				1		
OTU143_p_Actinobacteria_g_Jatrophihabitans	1				1		
OTU185_p_Actinobacteria_g_Arthrobacter	1				1		
OTU208_p_Actinobacteria_g_uncultured	1				1		
OTU212_p_Actinobacteria_	1				1		
OTU248_p_Actinobacteria_g_Actinoallomurus	1				1		
OTU252_p_Actinobacteria_	1				1		
OTU284_p_Actinobacteria_g_Patulibacter	1				1		
OTU286_p_Actinobacteria_g_Solirubrobacter	1				1		
OTU288_p_Actinobacteria_g_uncultured_bacterium	1				1		
OTU297_p_Armatimonadetes_g_Chthonomonas	1				1		
OTU327_p_Bacteroidetes_g_uncultured	1				1		
OTU348_p_Bacteroidetes_g_Ferruginibacter	1				1		
OTU351_p_Bacteroidetes_g_Flavisolibacter	1				1		
OTU357_p_Bacteroidetes_g_Parafilimonas	1				1		
OTU368_p_Bacteroidetes_g_uncultured_bacterium	1				1		
OTU387_p_Bacteroidetes_g_Mucilaginibacter	1				1		
OTU406_p_Chlamydiae_g_Candidatus_Metachlamydia	1				1		

(*continuation*)

OTU	pH	Ca	Mg	PCA1a	PCA1b	PCA2a	PCA2b
OTU407_p_Chlamydiae_g_Candidatus_Protochlamydia	1				1		
OTU100_p_Acidobacteria_	1					-1	
OTU103_p_Acidobacteria_g_uncultured_bacterium	1					-1	
OTU168_p_Actinobacteria_g_Intrasporangium	1					-1	
OTU204_p_Actinobacteria_g_Planosporangium	1					-1	
OTU231_p_Actinobacteria_g_Pseudonocardia	1					-1	
OTU263_p_Actinobacteria_Ambiguous_taxa	1					-1	
OTU276_p_Actinobacteria_g_uncultured_bacterium	1					-1	
OTU411_p_Chlamydiae_g_Candidatus_Rhabdochlamydia	1					-1	
OTU9_p_Thaumarchaeota_	1						
OTU16_p_Thaumarchaeota_g_uncultured_archaeon	1						
OTU27_p_Acidobacteria_Ambiguous_taxa	1						
OTU30_p_Acidobacteria_g_Candidatus_Koribacter	1						
OTU63_p_Acidobacteria_	1						
OTU84_p_Acidobacteria_Ambiguous_taxa	1						
OTU107_p_Acidobacteria_g_uncultured_bacterium	1						
OTU116_p_Actinobacteria_g_Candidatus_Microthrix	1						
OTU117_p_Actinobacteria_g_Iamia	1						
OTU139_p_Actinobacteria_g_Acidothermus	1						
OTU146_p_Actinobacteria_g_Blastococcus	1						
OTU165_p_Actinobacteria_	1						
OTU192_p_Actinobacteria_	1						
OTU198_p_Actinobacteria_g_Dactylosporangium	1						
OTU202_p_Actinobacteria_g_Micromonospora	1						
OTU235_p_Actinobacteria_g_Kitasatospora	1						
OTU237_p_Actinobacteria_g_Streptomyces	1						
OTU262_p_Actinobacteria_	1						
OTU265_p_Actinobacteria_g_uncultured_bacterium	1						
OTU266_p_Actinobacteria_g_uncultured_Rubrobacteridae	1						
OTU267_p_Actinobacteria_	1						
OTU271_p_Actinobacteria_g_uncultured_bacterium	1						
OTU287_p_Actinobacteria_	1						
OTU290_p_Actinobacteria_g_uncultured_bacterium	1						
OTU345_p_Bacteroidetes_g_uncultured_bacterium	1						
OTU363_p_Bacteroidetes_g_uncultured	1						
OTU405_p_Chlamydiae_	1						
OTU409_p_Chlamydiae_g_Parachlamydia	1						
OTU459_p_Chloroflexi_		-1		1	1		
OTU460_p_Chloroflexi_g_uncultured_bacterium		-1		1	1		
OTU473_p_Chloroflexi_		-1		1	1		
OTU480_p_Chloroflexi_g_Ktedonobacter		-1		1	1		
OTU915_p_Proteobacteria_g_uncultured_bacterium		-1					
OTU420_p_Chlorobi_g_uncultured_bacterium		1					

(*continuation*)

OTU	pH	Ca	Mg	PCA1a	PCA1b	PCA2a	PCA2b
OTU433_p_Chloroflexi_g_Roseiflexus				-1			
OTU441_p_Chloroflexi_Ambiguous_taxa				-1			
OTU696_p_Gemmatimonadetes_g_Gemmatimonas				-1			
OTU719_p_Nitrospirae_g_Nitrospira				-1			
OTU776_p_Proteobacteria_g_Phenylobacterium				-1			
OTU795_p_Proteobacteria_				-1			
OTU796_p_Proteobacteria_Ambiguous_taxa				-1			
OTU987_p_Proteobacteria_g_Novosphingobium				-1			
OTU453_p_Chloroflexi_g_uncultured_bacterium				1	1	-1	
OTU455_p_Chloroflexi_g_uncultured_bacterium				1	1	-1	
OTU461_p_Chloroflexi_				1	1	-1	
OTU807_p_Proteobacteria_g_uncultured				1	1	-1	
OTU809_p_Proteobacteria_Ambiguous_taxa				1	1	-1	
OTU820_p_Proteobacteria_g_uncultured_bacterium				1	1	-1	
OTU469_p_Chloroflexi_g_uncultured_bacterium				1	1		
OTU475_p_Chloroflexi_g_uncultured_bacterium				1	1		
OTU476_p_Chloroflexi_				1	1		
OTU758_p_Planctomycetes_g_Pir4_lineage				1	1		
OTU826_p_Proteobacteria_g_Pedomicrobium				1	1		
OTU836_p_Proteobacteria_g_uncultured_bacterium				1	1		
OTU541_p_Elusimicrobia_g_uncultured_bacterium					-1		
OTU456_p_Chloroflexi_					1	-1	
OTU812_p_Proteobacteria_g_Bradyrhizobium					1	-1	
OTU449_p_Chloroflexi_g_uncultured_bacterium					1		
OTU481_p_Chloroflexi_g_uncultured					1		
OTU483_p_Chloroflexi_g_uncultured					1		
OTU498_p_Chloroflexi_g_uncultured_bacterium					1		
OTU501_p_Chloroflexi_					1		
OTU514_p_Cyanobacteria_g_uncultured_bacterium					1		
OTU804_p_Proteobacteria_Ambiguous_taxa					1		
OTU839_p_Proteobacteria_g_Methylobacterium					1		
OTU940_p_Proteobacteria_g_uncultured						-1	
OTU760_p_Planctomycetes_g_Planctomyces							1
