# Supplementary figures and images for: Direct and indirect effects of a pH gradient bring insights into the mechanisms driving prokaryotic community structures
Source: Microbiome. 2018 Jun 11;6:106. doi: 10.1186/s40168-018-0482-8 (PMC5996553; doi:10.1186/s40168-018-0482-8)

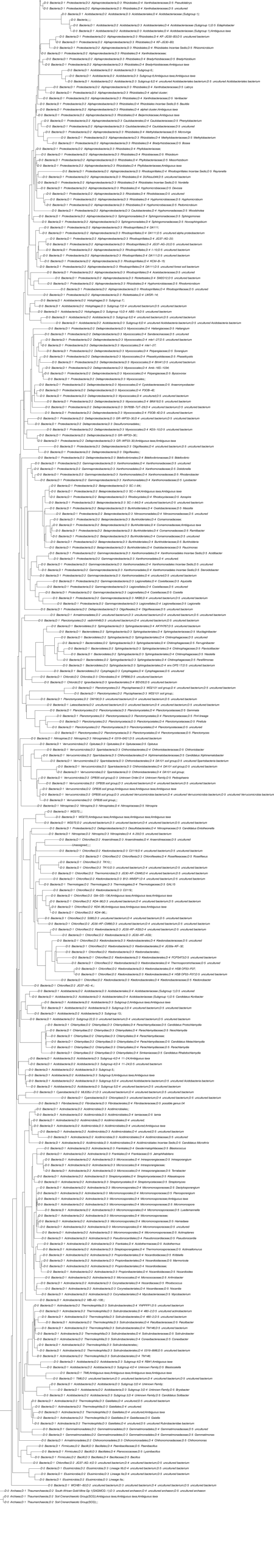

Supplement: Supplementary file 7 — Phylogenetic tree correspondent to the Figure 3 - HMSC models. (PDF 17 kb) [file 40168_2018_482_MOESM7_ESM.pdf]
